# Massive and rapid predominantly volcanic CO_2_ emission during the end-Permian mass extinction

**DOI:** 10.1073/pnas.2014701118

**Published:** 2021-09-07

**Authors:** Ying Cui, Mingsong Li, Elsbeth E. van Soelen, Francien Peterse, Wolfram M. Kürschner

**Affiliations:** ^a^Department of Earth and Environmental Studies, Montclair State University, Montclair, NJ 07043;; ^b^School of Earth and Space Sciences, Peking University, Beijing 100871, China;; ^c^Department of Geosciences, University of Oslo, Oslo 0371, Norway;; ^d^Department of Earth Sciences, Utrecht University, 3584 CB Utrecht, The Netherlands

**Keywords:** end-Permian mass extinction, compound specific carbon isotopes, CO_2_, Earth system model

## Abstract

The end-Permian mass extinction event (∼252 Mya) is associated with one of the largest global carbon cycle perturbations in the Phanerozoic and is thought to be triggered by the Siberian Traps volcanism. Sizable carbon isotope excursions (CIEs) have been found at numerous sites around the world, suggesting massive quantities of ^13^C-depleted CO_2_ input into the ocean and atmosphere system. The exact magnitude and cause of the CIEs, the pace of CO_2_ emission, and the total quantity of CO_2_, however, remain poorly known. Here, we quantify the CO_2_ emission in an Earth system model based on new compound-specific carbon isotope records from the Finnmark Platform and an astronomically tuned age model. By quantitatively comparing the modeled surface ocean pH and boron isotope pH proxy, a massive (∼36,000 Gt C) and rapid emission (∼5 Gt C yr^−1^) of largely volcanic CO_2_ source (∼−15%) is necessary to drive the observed pattern of CIE, the abrupt decline in surface ocean pH, and the extreme global temperature increase. This suggests that the massive amount of greenhouse gases may have pushed the Earth system toward a critical tipping point, beyond which extreme changes in ocean pH and temperature led to irreversible mass extinction. The comparatively amplified CIE observed in higher plant leaf waxes suggests that the surface waters of the Finnmark Platform were likely out of equilibrium with the initial massive centennial-scale release of carbon from the massive Siberian Traps volcanism, supporting the rapidity of carbon injection. Our modeling work reveals that carbon emission pulses are accompanied by organic carbon burial, facilitated by widespread ocean anoxia.

## Significance

The end-Permian mass extinction event (ca. 252 Mya) is the most-severe biodiversity loss in Earth’s history and is globally recognized by a rapid negative carbon isotope excursion. The trigger of this event, however, remains controversial. New paired terrestrial and marine compound-specific carbon isotope records may provide clues for this enigma. By comparing observed data to results from an isotope-enabled Earth system model, we find that a massive and rapid, predominantly volcanic CO_2_ emission during the Siberian Traps volcanism is likely the trigger for the carbon isotope excursion and the severe mass extinction. Our findings provide quantitative constraints of how a massive and rapid increase in CO_2_ may have influenced the marine ecosystem 252 Mya.

The end-Permian mass extinction (EPME) that occurred at 251.941 ± 0.037 Mya is considered the most severe biodiversity loss in Earth history (1, 2). The EPME coincides with the eruption of the Siberian Traps, a voluminous large igneous province (LIP) that occupies 6 million square kilometers (km^2^) in Siberia, Russia (3–5). The volcanic activity of this LIP is linked to SO_2_ and CO_2_ degassing generated by sill intrusion (6–10). The large amount of CO_2_ injected into the atmosphere is thought to have led to severe global warming (11–14), catastrophic ocean anoxia (15, 16), and extreme ocean and terrestrial acidification (17–21) being lethal for life on land and in the sea (22). To date, no agreement has been reached regarding the source of the ^13^C-depleted carbon that triggered the global carbon cycle perturbation, the decrease in ocean pH, and the global warming across the EPME. Additionally, atmospheric CO_2_ levels following the initial pulse of Siberian Traps volcanism and across the EPME remain poorly known (23, 24), limiting our understanding of the climate feedbacks that occur upon greenhouse gas release during this time.

**Fig. 1.**
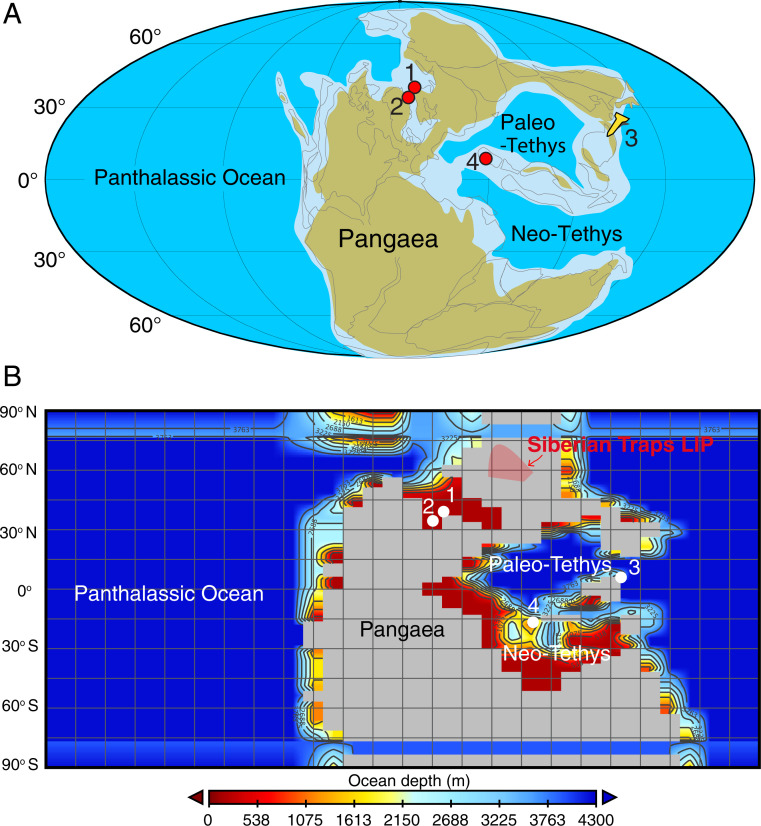
(*A*) Paleogeographical map of the Late Permian, with former and current coastlines. Indicated are 1) the location of Finnmark cores 7128/12-U-01 and 7129/10-U-01, 2) the East Greenland site at Kap Stosch discussed in ref. 52, 3) the GSSP site for the base of the Triassic at Meishan, China, and 4) the Kuh-e-Ali Bashi site of Iran (66, 107). The map was modified after ref. 61. (*B*) Paleogeography and paleobathymetry of the Late Permian used in cGENIE.

To address this critical gap in our knowledge, we constrain the source, pace and total amount of CO_2_ emissions using an Earth system model of intermediate complexity (i.e., carbon centric-Grid Enabled Integrated Earth system model [cGENIE]; *SI Appendix*) forced by new astronomically tuned δ^13^C records from well-preserved lipid biomarkers preserved in sediments from the Finnmark Platform, Norway. The Finnmark Platform is located offshore northern Norway on the Eastern Barents Sea shelf, hosting an expanded shallow marine section (paleo-water depth roughly 50 to 100 m) where two drill cores were collected (7128/12-U-01 and 7129/10-U-01) spanning the Permian–Triassic transition (Fig. 1). A previously generated bulk organic carbon isotope record (δ^13^C_org_) from the same core shows a two-step decline with a total carbon isotope excursion (CIE) magnitude of ∼4‰ (25). Although the sedimentary organic carbon was considered primarily of terrestrial origin, small contributions from marine organic carbon production could not be excluded. Here, we use compound-specific carbon isotope analysis of both long-chain and short-chain *n*-alkanes preserved in marine sediments in the Finnmark Platform to generate separate yet directly comparable records of δ^13^C for the terrestrial and the marine realm, respectively, across the EPME. Long-chain *n*-alkanes with a strong odd-over-even predominance (*n-*C_27_ and *n-*C_29_) are produced by higher plant leaf waxes, and their isotopic composition (δ^13^C_wax_) relates to their main carbon source (i.e., atmospheric CO_2_) (26). On the other hand, short-chain alkanes (*n-*C_17_ and *n-*C_19_) are derived from marine algae, and their δ^13^C values (δ^13^C_algae_) represent carbon in the marine realm (27, 28). To date, only a few EPME compound-specific carbon isotope studies have been reported, all of which are limited by unfavorable sedimentary facies or high thermal maturity of the organic matter (29, 30). In the present study, the exceptionally low thermal maturity of the organic matter is evident from the yellow color of pollen and spores, indicating a color index 2 out of 7 on the thermal alteration scale of Batten (31), which is equivalent to a vitrinite reflectance R_0_ of 0.3%. Moreover, the high sedimentation rate (discussed in *Carbon Cycle Quantification Using Astrochronology and Earth System Model*) of the siliciclastic sediments at the study site allows for studying both marine and terrestrial CIE across the EPME in unprecedented detail. Taken together, the Finnmark sedimentary records enable the reconstruction of individual yet directly comparable carbon isotope records for the terrestrial and the marine realm that can be astronomically tuned and used to quantitatively assess the source, pace, and total amount of ^13^C-depleted carbon released during the Siberian Traps eruption that led to the EPME. Using our new compound-specific carbon isotope records, rather than marine carbonates, has several advantages: 1) new astrochronology enables a 10^4^-year temporal resolution for our paired marine and terrestrial carbon isotope records; 2) we do not need to assume a constant sedimentation rate between tie point or using diachronous biozones to compare age like those used in global compilations (24) (see Fig. 4*A*); 3) the δ^13^C_algae_ data are not artificially smoothed as in ref. 32 to avoid underestimation of the CIE magnitude; and 4) our records are not affected by dissolution or truncation, a phenomenon common to shallow marine carbonates due to the presumed ocean acidification occurred during the EPME (18, 33). In addition, the directly comparable records of δ^13^C for the atmosphere and the ocean offer further insights into the size of the true CIE and rate and duration of carbon emissions.

## Results and Discussion

### Fidelity of the δ^13^C_algae_ and δ^13^C_wax_ Records.

**Fig. 2.**
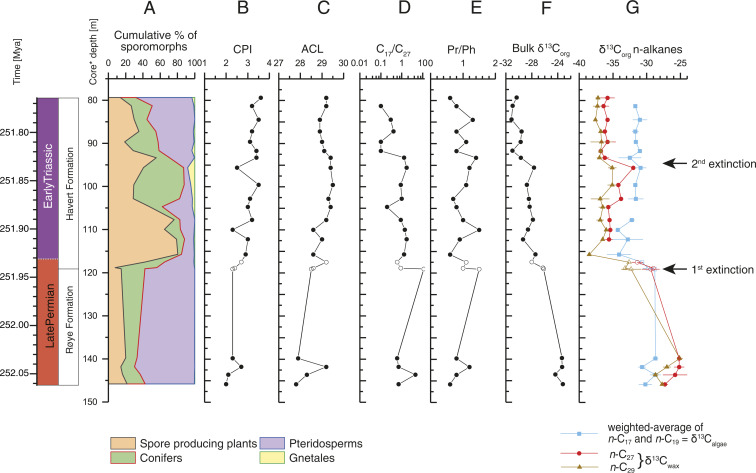
(*A*) Relative abundances of major groups of terrestrial palynomorphs. (*B*) Carbon preference index. (*C*) ACL of *n*-alkanes C_25_-C_33_. (*D*) Ratio between C_17_ and C_27_ *n*-alkane abundance. (*E*) Pristane/phytane (Pr/Ph) ratio. (*F*) Bulk organic δ^13^C values (δ^13^C_org_) measured in this study. (*G*) Compound-specific δ^13^C values of C_27_ and C_29_ *n*-alkanes (δ^13^C_wax_) and the weighted average of C_17_ and C_19_ *n*-alkanes (δ^13^C_algae_). Error bars represent the SD based on multiple analysis of the same sample. Individual values and SDs are given in *SI Appendix*, Table S6, and core depth refers to core 7128/12-U-01 (indicated by solid marks) with samples from 7129/10-U-01 (indicated by hollow marks) correlated as shown in *SI Appendix*, Fig. S2 and Table S5. The astronomically tuned age model (left) is detailed in Fig. 3.

The long-chain *n*-alkanes in the Finnmark core show a strong odd-over-even predominance as evidenced by a carbon preference index well above 2 (Fig. 2*B* and *SI Appendix*), supporting the presumed low thermal maturity of the sediments (34). The average chain length (ACL_C25-C33_) varies between 27.5 and 29.5 and shows an increasing trend up-core (Fig. 2*C*), in which higher ACL values correspond to high numbers of conifer pollen counts in the same core (e.g., at 110- to 95-m core depth), and low ACL values are associated with the pteridosperm (seed fern)-dominated ecosystem that existed prior to the EPME (Fig. 2*A*). The interval with intermediate ACL (116- to 108-m core depth) is linked to a spore peak and the first negative excursion in the δ^13^C record of sedimentary organic matter (δ^13^C_org_) (Fig. 2*F*), suggesting that local terrestrial ecosystem reorganization may have modified the original δ^13^C_org_ signal across the EPME at this site (25, 35). Short-chain *n*-alkane abundances show more variation throughout the section and are sometimes dominant over the longer chain *n*-alkanes, indicative of changing marine and terrestrial contributions to the total carbon pool (Fig. 2*D*).

In addition to δ^13^C_org_ (Fig. 2*F*), we report the δ^13^C_algae_ as the weighted-average of *n-*C_17_ and *n-*C_19_ alkane δ^13^C values and δ^13^C_wax_ as the weighted-average of *n-*C_27_ and *n-*C_29_ alkane δ^13^C values (Fig. 2*G*). The δ^13^C_algae_ record displays a two-step negative CIE, of which the first exhibits a magnitude of ∼5‰ and the second a magnitude of ∼1.5‰ (Fig. 2*G*), similar to the record of δ^13^C_org_ (∼5‰ and ∼2‰ during the two negative CIEs, respectively) (Fig. 2*F*). The δ^13^C_wax_ shows a similar trend to the δ^13^C_algae_ record during the two negative CIEs (Fig. 2*G*), although the magnitude is twice that in δ^13^C_algae_. Such terrestrial CIE amplification is also seen from several other Permian-Triassic boundary (PTB) sites in China (24, 36), Australia, and Antarctica (37). The amplified CIE magnitude in δ^13^C_wax_ may be partly attributed to rising atmospheric CO_2_ levels (38) and augmented by changes in terrestrial vegetation types and hydrological cycle (39). However, a plausible alternative interpretation of the amplified terrestrial CIE signal is that the surface waters of the Finnmark platform were out of equilibrium with the rapid release of carbon from the massive volcanism. The astronomically tuned age model (see *Materials and Methods*) allows for detailed correlation of the two negative CIEs in the biomarkers: the first CIE is associated with the EPME between 251.941 ± 0.037 Mya and 251.880 ± 0.031 Mya (1), and the second CIE appears to immediately predate the second extinction pulse at 251.761 ± 0.06 Mya (40) (see Fig. 5 and in *SI Appendix*). The onset of the first CIE in δ^13^C_algae_ occurs at 119.1-m core depth (Fig. 2, 251.941 Mya or time 0 in our model simulation) (1), and the δ^13^C_algae_ rapidly declines to −34.1‰ at 115.7 m (251.926 Mya) followed by a small rebound to −32.8‰ at 112.2 m (251.910 Mya) before reaching a minimum value of −34.3‰ at 110.0 m (251.900 Mya). If we define the CIE onset as the most rapid decline in δ^13^C_algae_, then the CIE onset duration is calculated to be 15 Kyr (from 251.941 to 251.926 Mya). The δ^13^C_algae_ remain >2‰ lower than the pre-CIE values, while both δ^13^C_org_ and δ^13^C_wax_ stay >6‰ lower than their pre-CIE values, suggesting changes in terrestrial vegetation types and hydrological cycle persisted in the terrestrial realm regionally.

**Fig. 3.**
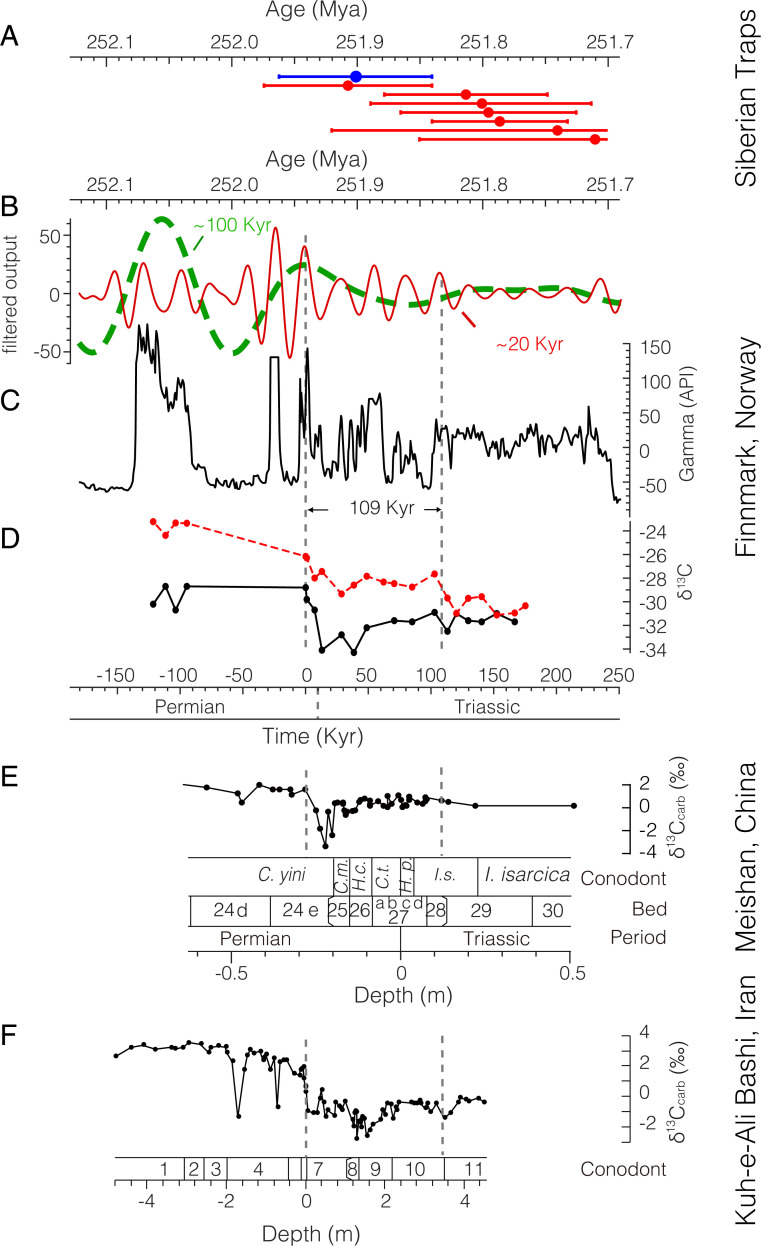
Time series analysis. (*A*) Weighted mean uranium-lead (U-Pb) dates reported with 2σ analytical uncertainties for Siberian Traps LIP sills (red) and pyroclastic rocks (blue) (5). (*B*) ∼100-Kyr eccentricity (dashed green) and 20-Kyr precession (red) Gauss bandpass-filtered cycles (passband is 0.0085 ± 0.0025 and 0.045 ± 0.01 cycles/Kyr, respectively). (*C*) Time-calibrated gamma ray series in American Petroleum Institute (API) unit from cores 7128/12-U-01. (*D*) Time-calibrated δ^13^C_org_ (red) and δ^13^C_algae_ (black) across the Permian–Triassic transition. (*E*) δ^13^C_carb_ record for the Meishan section, China (2) shown with bed numbers and conodont zones (108). Conodont zones: *C. m.*=*Clarkina meishanensis*, *H. c.* = *Hindeodus changxingensis*, *C. t.* = *C. taylorae*, *H. p.* = *H. parvus*, and *I.s.* = *Isarcicella staeschei*. (F) δ^13^C data at Kuh-e-Ali Bashi section of Iran (66, 107) shown with conodont zones (109, 110): 1) *C. changxingensis*, 2) *C. bachmanni*, 3) *C. nodosa*, 4) *C. yini*, 5) *C. abadehensis*, 6) *C. hauschkei*, 7) *H. praeparvus-H. changxingensis*, 8) *M. ultima-S. ?mostleri*, 9) *H. parvus*, 10) *H. lobota*, and 11) *I. staeschei*.

**Fig. 4.**
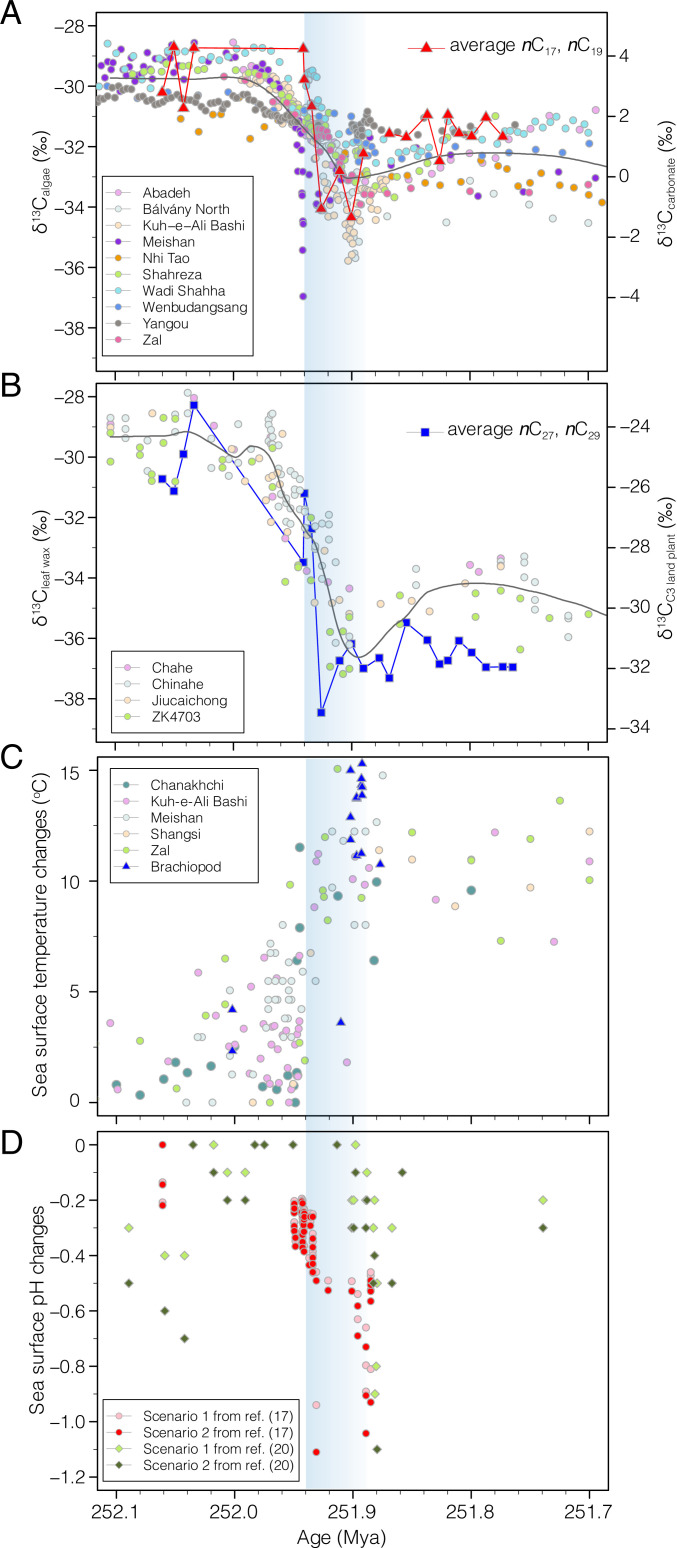
Synthesized proxy records of carbon isotopes from marine carbonates and fossil C_3_ land plants remains, sea surface temperature, and pH. (*A*) Comparison between δ^13^C_algae_ and global marine carbonate carbon isotopes from sites at Abadeh, Kuh-e-Ali Bashi, Shahreza, and Zal in Iran, Meishan, Wenbudangsang, and Yanggou in South China, at Bálvány North in Hungary, and at Nhi Tao in Vietnam (24). (*B*) Comparison between δ^13^C_leaf_ wax and the δ^13^C of sedimentary leaf cuticles and wood of C_3_ land plants from South China (24). (*C*) Reconstructed sea surface temperature data using conodont fossils (circles) (24) and brachiopods (triangles) (14). The conodont-based temperature data are from sites in the Paleo-Tethys, including Chanakhchi, Kuh-e Ali Bashi, Meishan, Shangsi, and Zal. (*D*) Relative changes in sea surface pH based on boron isotope proxy from ref. 17 and ref. 20. Pink and red circles are data from scenario 1 and scenario 2 in ref. 17, and green and blue diamonds are data from scenario 1 and scenario 2 in ref. 20.

The δ^13^C_algae_ record is considered representative of the ocean dissolved inorganic carbon (DIC) system across the EPME for several reasons. Two factors that may have affected the marine δ^13^C_algae_ record, marine anoxia, and changes in microbial communities (e.g., ref. 41) do not play a role on the Finnmark Platform. Firstly, the widespread interval of anoxic conditions that developed in many locations that may have decreased the δ^13^C_org_ values (25, 42) during the EPME (15, 43–48) is not observed in the Finnmark cores because of low-density bioturbation (49, 50) and the absence of amorphous organic matter (50, 51). This is in agreement with pristane/phytane (Pr/Ph) ratios being greater than 1 for most of the samples (Fig. 2*E*). Only short intervals with dysoxic conditions have been identified (50), which correlate with Pr/Ph ratios of 0.6 at 116 m, 103 m, and 90 m. However, the δ^13^C_org_ and δ^13^C_algae_ records do not show a consistent correlation with the temporary dysoxic conditions indicated by low Pr/Ph values (*SI Appendix*). Because of this rather-stable marine depositional environment, we presume that the composition of the marine microbial community remained unchanged. This is a plausible scenario as hopane/sterane ratios over the Permian–Triassic boundary interval at Kap Stosch on East Greenland (Fig. 1, location 2) are fairly stable around 1.5 to 2 in a similar depositional setting (52). Similar to terrestrial higher plants, carbon isotope fractionation of marine algae can also be sensitive to changes in atmospheric CO_2_ (53, 54). The increased CO_2_ may have amplified the Finnmark algae biomarker, but the ocean biogeochemistry may have muted such effect and exerts little net change to its overall CIE signal (Fig. 2*F*). Nevertheless, the magnitude of the CIE for δ^13^C_algae_ remains similar to the range of 4 to 5‰ reported from the Paleo-Tethys marine carbonate (55) (Fig. 3). In order to better understand the effects of large-scale CO_2_ release on climate and environmental conditions and thus identifying the exact cause of the extinction, it is crucial to constrain the magnitude of the excursion to estimate the rate and magnitude of CO_2_ release (23, 32, 56). Many published Late Permian δ^13^C records for the marine realm are based on bulk carbonates (δ^13^C_carb_), most of which are from carbonate platforms in the Paleo-Tethys Ocean (55). One potential bias in the marine carbonate records is the sediment mixing that can have an effect on the δ^13^C_carb_, as shown for deep-sea sections during the Paleocene-Eocene Thermal Maximum (PETM) (57). Additionally, some of these carbonates may be highly condensed due to extremely low accumulation rates (2, 58), which makes it difficult to distinguish the EPME from the Permian–Triassic boundary (1, 59). For example, the Meishan Global Boundary Stratotype Section and Point (GSSP) section in South China (Fig. 1, location 3) is impacted by extremely low sedimentation rate (<1 cm Kyr^−1^), but the magnitude of CIE (∼4 to 5‰) is similar to that in δ^13^C_algae_ from this study. On the other hand, one of the most expanded carbonate sections, the Kuh-e-Ali Bashi section in Iran (Fig. 1, location 4 and Fig. 3*F*), shows several negative isotope excursions in δ^13^C_carb_ record of the Late Permian and Early Triassic (60), during which the magnitude of the CIE associated with the EPME is about 4‰, similar to the CIE magnitude observed in the δ^13^C_algae_ record reported here (Fig. 3), another line of evidence supporting that δ^13^C_algae_ is representative of the ocean DIC. In contrast, both δ^13^C_wax_ data and δ^13^C_plant_ data from cuticles and wood of C_3_ land plant remains in South China (24) show augmentation in their CIE magnitude compared to δ^13^C_algae_ and δ^13^C_carb_ (Fig. 4 *A* and *B*). Therefore, we suggest that the *n*-alkanes in the Finnmark section are faithful recorders of the global surface ocean carbon cycle perturbation resulting from their pristine preservation. Although our sampling resolution is lower than that of existing bulk marine carbonate records, our new compound-specific carbon isotope records are the highest of their kind for this time interval and enable us to separate terrestrial and marine signals (Fig. 4 *A* and *B*). In addition, our records are more suitable for the isotope inversion modeling because the astrochronology allows for the age of each sample to be very well constrained. In contrast, despite the high temporal resolution for marine bulk carbonate records, the age of these samples is only interpolated from lithostratigraphy or biostratigraphy, which makes the high sampling resolution less relevant. Additionally, the high sedimentation rates of the siliciclastic sediments at the Finnmark Platform provide us with the high-quality records required to establish high-fidelity geochronology based on cyclostratigraphy (61).

### Carbon Cycle Quantification Using Astrochronology and Earth System Model.

To quantify the carbon cycle perturbations across the EPME, we constructed a high-resolution astrochronological age model for the Finnmark core. Time series analysis of the total gamma ray intensity at core 7128/12-U-01 provides a floating astronomical time scale for the carbon isotope records (*SI Appendix*, *Materials and Methods*). The power spectrum of the gamma ray series shows that the dominant cycles are at 23 m, 7.4 m, 5.5 m, 4 m, and 2.7 m (*SI Appendix*, Figs. S6–S8). The evolutionary power spectrum indicates that the 23-m cycles represent the dominating cyclicity throughout the series and that the 5- to 7-m cycles are the leading cyclicities across the Permian–Triassic boundary interval (ca. 125- to 105-m core depth; *SI Appendix*, Fig. S9). Correlation Coefficient and time scale optimization analyses suggest that the optimal mean sedimentation rate is ∼22.4 cm/Kyr (*SI Appendix*, Figs. S10 and S11), which enables generating a 432-Kyr-long floating astrochronology for the Finnmark core (Fig. 3). The initial CIE is correlated with the EPME onset from the GSSP at Meishan, South China (1) (*SI Appendix*, Figs. S13 and S14) and is set to a 251.941 ± 0.037 Mya age (Meishan) (1) for the construction of an absolute astronomical time scale. The Finnmark Platform CIE duration (115.7- to 93.3-m core depth) encompasses approximately one eccentricity cycle (Fig. 3*A*), which is comparable to the Meishan GSSP for the basal Triassic (*SI Appendix*, Fig. S14). Cyclostratigraphy of the Meishan uranium series suggests that the two pulses of extinction lasted for about 40 Kyr, and the largest CIE lasted for about 6 Kyr (61). Other estimations of the EPME interval at Meishan are 60 ± 48 Kyr based on uranium–lead (U-Pb) dating (1) and 83 Kyr based on cyclostratigraphy (62). However, the highly condensed nature of the Meishan section limits the confidence of its astrochronology. Here, the high sedimentation rate (22.4 cm/Kyr versus <1 cm/Kyr at Meishan and Daxiakou of South China) (61) associated with continuous deposition provides a more reliable estimation of the CIE duration of 109 Kyr and a CIE onset duration of 15 Kyr. This slightly longer duration of the end-Permian CIE compared to previous estimates implies that the carbon cycle within the ocean and atmosphere system has a longer time to respond and recover from perturbation. The end-Permian CIE began at the peak of a 100-kyr cycle at both the Finnmark Platform and the GSSP Meishan sections (*SI Appendix*, Fig. S14), providing additional evidence for the global correlation of the carbon isotope records.

**Fig. 5.**
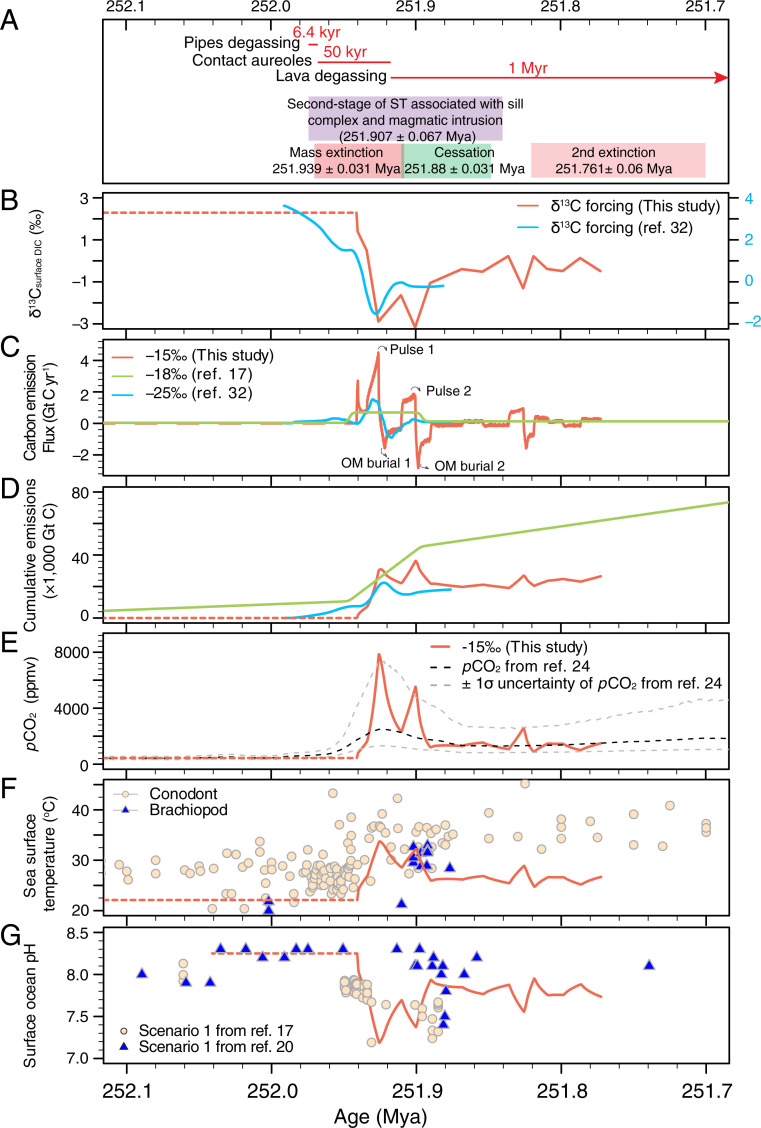
Key model results from the RMSE-determined best fit. (*A*) Links between the EPME and the timing of Siberian Traps volcanism. Age of mass extinction is 251.939 ± 0.031 Ma based on Shen et al. (106) and age of second-stage of Siberian Traps volcanism is 251.907 ± 0.067 Ma from Burgess et al. (4) Duration of pipe degassing, contact aureoles, and lava degassing are from Svensen et al. (9), assuming that the onset age is 251.907 ± 0.067 Ma. Also shown is the age of the extinction cessation at 251.88 ± 0.031 Ma based on Meishan Bed 28 (5) and the age of second extinction in the earliest Triassic at 251.761 ± 0.06 Ma (40). Note that the red curve is based on δ^13^C_algae_ assuming a constant fractionation between algae and DIC of 31‰, which is within the range of maximum fractionation for marine algae (111–113). (*B*) δ^13^C forcing comparison of the surface DIC for this study and (32) derived from the GSSP Meishan section after loess curve fitting. (*C*) Modeled carbon emission rate in Gt C yr^−1^ from the best-fit scenario (red) and comparison to the carbon emission rate for the organic matter scenario in (32) (blue) and Jurikova et al. (17) (green). (*D*) Modeled cumulative carbon emission in Gt C from the best-fit scenario (red) and comparison to the carbon emission rate for the organic matter scenario in Cui et al. (32) (blue) and Jurikova et al. (17) (green). (*E*) Modeled changes in atmospheric *p*CO_2_ in ppmv from the best-fit scenario (red) and the reconstructed continuous *p*CO_2_ from Wu et al. (24) based on carbon isotopes of fossil C_3_ plant remains. (*F*) Modeled changes in global sea surface temperature in °C from the best-fit scenario (red) and comparison to reconstructed Paleo-Tethys ocean temperature based on δ^18^O of well-preserved conodonts (beige circles) (24) and brachiopods (blue triangles) (14). (*G*) Modeled surface ocean pH decline from the best-fit scenario (red) and comparison to the boron isotope proxy pH reconstruction from Jurikova et al. (17) and Clarkson et al. (20). Dashed red lines in *B–G* represents the steady-state condition from the 200-Kyr-long spin-ups.

Previous modeling work inverted the δ^13^C record of marine carbonate from the GSSP Meishan section (32, 63), which is impacted by the highly condensed nature of the site (2). Inverting δ^13^C alone also suffers from nonunique solutions when determining the carbon emission flux by assuming the isotopic composition of an unknown source (32, 63). We improved upon previous work by using the surface ocean pH records (17, 20) and sea surface temperature records (14, 64–66) as additional constraints to determine the most-plausible carbon source and associated carbon emission rate by minimizing the root mean square root (RMSE) (*SI Appendix*). This approach allows for the determination of the best-matched δ^13^C value of the CO_2_ emitted and therefore quantifying the paleoclimatological effects of this important greenhouse gas. Using the newly constructed astrochronology in the Finnmark Platform, the pace of CO_2_ changes across the EPME is estimated via carbon isotope inversion based on δ^13^C_algae_ along with the age model described above using cGENIE model and further constrained by δ^11^B as a proxy of pH (17) and δ^18^O as a proxy of temperature. The initial and boundary conditions of the end-Permian are adapted from ref. 32, except that the initial *p*CO_2_ is set to be 440 ppmv following recent studies by Li et al. (67) using the stomatal ratio and Wu et al. (24) using the δ^13^C of fossil C_3_ plant remains. We ran simulations that consider a broad combination of model parameters and assumptions associated with proxy interpretation (*SI Appendix*, Tables S1 and S2). Our model ensemble includes seven simulations (*SI Appendix*, Table S3), each associated with a unique isotopic signature of the carbon source forced by the compound-specific carbon isotope records of marine algae and the astrochronology generated in this study (Figs. 3 and 4). Based on the smallest RMSE between our modeled pH and the proxy records, we identify that the best-matched scenario is associated with δ^13^C = −15‰ (refer to *SI Appendix* for the RMSE results). This best-fit scenario is associated with a large pulse of mainly volcanic CO_2_ from the Siberian Traps volcanism (53% if we assume the δ^13^C of peridotite-derived CO_2_ is −6‰ as in ref. 68 and 77% if the δ^13^C of recycled crust CO_2_ is −12‰ as in ref. 69), if the remaining CO_2_ is derived from sedimentary organic matter oxidation (9) or coal combustion (70). This scenario is associated with a massive amount of total CO_2_ emission (approaches ∼36,000 Gt C) over the 168 Kyr simulation duration and a maximum emission rate of 4.5 Gt C yr^−1^ (Fig. 5), roughly half the size of current carbon emission rate (71, 72). The atmospheric *p*CO_2_ increases from ∼440 (67) to ∼7,390 ppm when δ^13^C_algae_ reaches its first minimum, with the maximum *p*CO_2_ value falling in the upper range of a recent CO_2_ reconstruction using an independent estimate based on carbon isotopes of fossil C_3_ land plant remains across the EPME (24). This 13× increase in atmospheric CO_2_ causes global temperature to rise from 25 to 40 °C, in accordance with temperature proxy data based on well-preserved brachiopod shells (13, 14) but slightly smaller than the temperature proxy data using conodonts (64–66), suggesting that the δ^13^C_source_ may have become higher than −15‰ during the warmest interval, consistent with varying δ^13^C_source_ with evolving degassing style. The CO_2_-driven warming response of the global ocean likely differ due to changes in the ocean circulation pattern. Our model results show extreme warmth in the eastern Paleo-Tethys relative to in the western Paleo-Tethys (*SI Appendix*, Fig. S25), providing spatial variability that can be further compared with temperature proxy data for the eastern and western Paleo-Tethys (11), a feature that cannot be revealed in the one-dimensional biogeochemical box model used in (17). Intriguingly, our preferred δ^13^C_source_ of −15‰ coincides with an independent estimate based on a calcium isotopic mass balance model (19) and lies within the range (−11 to −17‰) suggested by Gutjahr et al. (73) for the PETM, indicating the two events share similar trigger mechanisms. Indeed, the PETM has been linked to the North Atlantic Igneous Province with a volume as large as 150,000 km^3^ (74). It is important to note that the PETM is only associated with a small extinction of the benthic foraminifera due to the presumed slow rate of carbon emission (75, 76). In the following, we compare the carbon emission history of the EPME based on our inverse modeling and results from the literature.

The simulated CO_2_ emission exhibits at least two pulses that appear to be global (pulse 1 and 2) (*SI Appendix*). Prior to the main emission pulse, a small amount of carbon was emitted at a peak rate of 2.7 Gt C yr^−1^ (average rate 1.6 Gt C yr^−1^) that lasted for 1.9 Kyr (maintaining emission rate at > 1 Gt C yr^−1^), which reflects a near-instantaneous drop in δ^13^C_algae_ during the CIE onset. This initial emission pulse is not seen in the previous modeling work (17, 32) (Fig. 5*C*) and therefore may be an artifact from local effects. The two major CO_2_ emission pulses (labeled pulse 1 and pulse 2) are associated with a maximum carbon emission rate of 4.5 Gt C yr^−1^ and 1.9 Gt C yr^−1^ (average 2.6 Gt C yr^−1^ and 1.5 Gt C yr^−1^) and lasted for 8.3 Kyr and 9.1 Kyr respectively (maintaining emission rate at > 1 Gt C yr^−1^). The largest emission pulse (pulse 1) is two times larger than the estimate from a previous model inversion (32) using the GSSP Meishan carbon isotope record (Fig. 5*A*) and 7× as large as the estimate in ref. 17 (0.7 Gt C yr^−1^) based on a forward biogeochemical model. This discrepancy likely resulted from the smaller magnitude of the CIE recorded in shallow marine carbonate sections, the lack of astrochronology, and a lower assumed δ^13^C_source_ in these previous studies (17, 32). The model results from slower emission rate and lower *p*CO_2_ (∼4,400 ppm) in Jurikova et al. (17) did not capture the full magnitude of the proxy estimate of pH decline and temperature rise (their Fig. 2 *C* and *H*) and therefore higher emission rate and *p*CO_2_ are required to better match the proxy data. The two main pulses appear to overlap in timing with the intrusive phase of the Siberian Traps volcanism (4, 8, 9) (Fig. 5 and *SI Appendix*, Table S5) following the onset of sill complex and magmatic intrusion (251.907 ± 0.067 Mya) (4), which supports a direct link between the Siberian Traps volcanism and the CO_2_ emission (Fig. 5). The degree of warming (>10 °C) and acidification (∆pH ∼1), the CIE magnitude (∼5‰), and the CIE onset duration (15 Kyr) dictate that the carbon emission rate during the peak EPME is up to 7× faster than that during the PETM (0.6 Gt C yr^−1^ in ref. 73 and 1.7 Gt C yr^−1^ in ref. 75). The final carbon emission pulse lasted longer (10.3 Kyr) but has a much smaller peak emission rate of 1.0 Gt C yr^−1^ (average 0.8 Gt C yr^−1^), although it is not seen in previous work and may be a local signal (Fig. 5*C*). In addition to the rapidity of the carbon injection during the peak EPME, the cumulative carbon emission (36,200 Gt C) amounts to 3× that across the PETM (10,200 to 12,200 Gt C) (Fig. 5*D*) and may have been the ultimate driver of the much-more-severe ecological consequences at the end of the Permian Period. The ∼36,000 Gt C cumulative carbon added in the first 15,000 y of our simulation is well within the range of estimated total emission from phreatomagmatic pipe degassing, contact aureoles, and lava degassing from the Siberian Traps volcanism (9, 74, 77) (*SI Appendix*) and similar to an estimate from ref. 17, although these authors allow the mantle-dominated carbon emission to continue following the CIE. Nevertheless, we find that there is large uncertainty associated with the total degassing budget from the Siberian Traps volcanism and that the δ^13^C_source_ values could deviate from −15‰ depending on the eruption style. Indeed, the δ^13^C_source_ value may become higher through the emission period, with more mantle carbon later on during Siberian Traps emplacement. More mantle carbon would have led to higher *p*CO_2_ modeled here and could explain the low pH values observed following “pulse 2” in Fig. 5. Besides the emission from the degassing from the Siberian Traps, rapid degradation of labile organic carbon from rapidly accumulating organic matter in the ocean (78) and from catastrophic terrestrial soil erosion (79) may have augmented the carbon emission flux. Each carbon emission pulse is followed by negative fluxes necessary to explain the carbon isotope recovery (maximum carbon sequestration following pulse 2, at −2.8 Gt C yr^−1^), which indicate organic carbon burial in the ocean (Fig. 5). Increased organic carbon burial is facilitated by the widespread ocean anoxia (15, 16, 80) and high phosphate concentration resulting from increased continental weathering that promoted primary productivity in the surface ocean (81, 82).

Similar to nutrient levels, the alkalinity of the ocean is expected to increase due to increased silicate weathering rate after a large pulse of CO_2_ emission (83). Freshly erupted basalt from the Siberian Traps volcanism may have accelerated the CO_2_ consumption. However, the eruption emplaced volcanic rocks into areas on the Earth’s surface where weathering rates are relatively low, and the expected continental weathering increase would be acting on preexisting transport-limited areas of the Earth’s surface (84–89). This limited effect of CO_2_ drawdown may have led to a failure of the silicate weathering and extended the warming well into the Early Triassic (90). As an important caveat, the model does not consider explicitly expressed lithology, despite the inclusion of a temperature-dependent silicate weathering feedback. We note a 2.7× increase in the weathering flux (30 Tmol yr^−1^ to 79 Tmol yr^−1^) from the elevated CO_2_ and temperature (*SI Appendix*), but the increased weathering flux did not drive a full recovery in *p*CO_2_ and temperature, suggesting the temperature-dependent silicate weathering feedback alone is insufficient to recover the climate system within the model simulation duration. The increased weathering flux led to higher alkalinity in the surface ocean, which increases from 4.5 to 5.3 mmol kg^−1^ when the maximum alkalinity is reached at 41 Kyr from the CIE onset. The surface ocean pH declined by 1.1 unit from 8.3 to 7.2, which is consistent with the amount of pH decline based on boron isotope proxy from well-preserved brachiopods in the Southern Alps of northern Italy (17) (0.9 to 1.1 unit decline for the two scenarios associated with whether vital effects affect the δ^11^B-pH dependency) and selected micrite and early cemented grainstones from the Musandam Peninsula in United Arab Emirates (20) (0.9 to 1.1 unit decline assuming −34 and −36.8‰ δ^11^B_seawater_ value). It should be noted that these pH change estimates are likely conservative if ancient calcifying brachiopods have larger vital effects than assumed in these studies. Higher *p*CO_2_ also resulted in lower benthic oxygen levels, which reduced significantly from 152 μmol kg^−1^ to 0 μmol kg^−1^ at ∼9.9 Kyr from the CIE onset. The cumulative carbon emission reached ∼30,800 Gt C following emission pulse 1, which corresponds to maximum *p*CO_2_ (7,844 ppmv) and coincides with the maximum modeled temperature (33.7 °C), similar to the proxy record based on oxygen isotope from well-preserved brachiopod shells (33 °C; Fig. 5 *B*–*D*). Emission pulse 2 was followed by a second peak of *p*CO_2_ (5,530 ppmv) and temperature (32.2 °C). The lowest pH also coincides with the emission pulse 1, underwriting the importance of a rapid CO_2_ emission associated with the Siberian Traps volcanism. This model inversion strongly supports that it is the rapid and massive CO_2_ that led to the abrupt decline in pH, extreme increase in surface ocean temperature, and the combined environmental effects that drove the mass extinction at 251.939 ± 0.031 Mya. The transition from mainly flood lavas to predominantly sill intrusions during the Siberian Traps volcanism (4) coincide with the onset of the global CIE and the EPME, providing a valid trigger mechanism for the CO_2_ release from volcanic CO_2_. Elevated mercury levels across the EPME in sections found in many locations, including China (91, 92), Arctic Canada (93, 94), and the United States (95), are supportive of the volatile gas emissions during sill intrusion and lava degassing. Recent mercury mass balance modeling provides additional quantitative constraints to the mercury loading related to volcanic eruption and sill intrusion (96, 97). The largely volcanic CO_2_ scenario presented here, if correct, can provide fundamental implications to understand the Earth system response to the large pulse of CO_2_ emission and the relationship between rapid climate change and mass extinction through time.

In addition to the paleoclimate implications from the modeling study on carbon emission, we can gain further insight on the controls of the CIE amplification in δ^13^C_wax_. This amplification could be attributed to three factors: 1) an increase in atmospheric CO_2_ levels (38); 2) changes in hydrological conditions and vegetations (39); and 3) a disequilibrium between the surface ocean and the atmosphere (98). The amount of amplification due to increased *p*CO_2_ is highly dependent on the initial *p*CO_2_ prior to the EPME (i.e., a lower initial *p*CO_2_ will yield a larger photosynthetic fractionation and vice versa). The ∼440-ppm CO_2_ level reconstructed from stomatal proxies (67) may underestimate the *p*CO_2_ in the latest Permian (23), which could lead to an overestimation of the increase in photosynthetic fractionation. If the initial atmospheric *p*CO_2_ was higher than that indicated by stomatal proxies (i.e., ∼2,800 ppm as in ref. 32), the increase in photosynthetic discrimination due to increased *p*CO_2_ is only ∼1‰ (*SI Appendix*), which allows for an alternative interpretation for the amplified terrestrial CIE. Terrestrial plant leaf waxes directly sample the atmosphere reservoir, which suggests that the atmosphere changed much more than what is observed in the equilibrium ocean response, implying that the change may have occurred faster than the equilibration time between these reservoirs during the EPME. A larger atmospheric CIE than that in the surface waters is observed for a very-rapid carbon release scenario for the PETM in Kirtland Turner and Ridgwell (99) in cGENIE. Such an alternate possibility suggests that the surface waters of the Finnmark platform were out of equilibrium with the initial massive centennial-scale release of carbon as a result of the massive Siberian Traps volcanism. A significantly larger atmospheric CIE magnitude during the PTB implies the carbon emission is rapid and massive, which is consistent with our modeling results. A critical rate of the Earth’s carbon cycle perturbation may have been reached, which triggered magnifying feedbacks and ultimately drove the Earth’s system beyond a threshold and lead to the mass extinction (100, 101).

## Conclusions

Late Permian shales and siltstones from the Finnmark Platform are ideal for compound-specific isotope analyses and the subsequent carbon cycle quantification, as the organic matter is well preserved and marine environmental conditions remained stable. The resulting compound-specific carbon isotope records reveal a first, simultaneous negative shift in δ^13^C_algae_ and δ^13^C_wax_ of 4 to 5‰ and 10 to 11‰, respectively. This first negative CIE in the Finnmark δ^13^C records is linked to the globally recognized negative shift that marks the EPME, while the second negative CIE immediately predates the second small-scale extinction in the earliest Triassic (Fig. 5). The high sedimentation rate associated with continuous deposition in the Finnmark core enables a more reliable astrochronological estimation of the CIE onset duration of 15 Kyr and a total CIE duration of 109 Kyr. The high-resolution δ^13^C_algae_ record presented in this study is similar in shape to the global CIE seen elsewhere (Fig. 4 *A* and *B*) in that they show similar CIE magnitude and exhibit multiple negative CIE pulses. An Earth system model of intermediate complexity with realistic continental configuration and ocean bathymetry for the latest Permian conditions was used to simulate the carbon emission history using carbon isotope inversion of the δ^13^C_algae_ records. Our best-fit model δ^13^C_source_ value is determined be close to −15‰ by minimizing the root mean square error between modeled pH and boron isotope pH proxy estimates and between modeled sea surface temperature and oxygen isotope temperature proxy records. This suggests that atmospheric *p*CO_2_ increases by 13× pre-extinction level with at least two separate pulses at a maximum rate of 4.5 Gt C yr^−1^, each corresponding to the emplacement of the Siberian Traps volcanism, consistent with a largely volcanic source (53 to 77%) with smaller contributions from coal combustion or thermogenic methane during sill intrusion. Our work also highlights the air–sea disequilibrium evidenced by the significantly amplified CIE of the atmosphere, which supports our major conclusion of a rapid and massive CO_2_ emission. Temperature-dependent silicate weathering feedback and organic carbon burial is insufficient to drive the full carbon isotope recovery to the pre-extinction level, with atmospheric *p*CO_2_ remains at >3× larger than pre-event level, leading to the long-term Early Triassic warmth. Taken together, we suggest that it is the rapid and massive amount of largely volcanic CO_2_ emission and associated feedbacks that led to the catastrophic mass extinction. It should be noted that despite the new astrochronology-based, compound-specific carbon isotope records and the advanced Earth system modeling results in the present study, there are still some unresolved questions that need to be addressed in future studies. Foremost, we would like to mention here 1) the chronology of the individual volcanic pulses and their exact correlation with the δ^13^C shifts in the sedimentary records, 2) the links between the evolution of δ^13^C_source_ and pulsed CO_2_ degassing history, which is dependent on host-rock properties, and 3) that caution is warranted when interpreting the structure of carbon isotope records from a single site due to low density of data. Hence, future modeling efforts need a denser collection of globally representative ocean and atmosphere carbon isotope data with an astronomically tuned age model to produce an even more reliable inversion and to compare with the current results.

## Materials and Methods

### Material.

Details on the lithology and stratigraphy can be found in *SI Appendix* and ref. 49. In short, samples were collected from two parallel cores from three different units: an Upper Permian siltstone unit, the top of a Late Permian wackestone/grainstone unit existing of pebbly shale and in some places phosphorite, and a Lower Triassic siltstone/shale unit. A total of 19 samples were selected for lipid extraction from core 7128/12-U-01, and three samples were added from core 7129/10-U-01. Samples were taken from dark, organic rich shale/siltstone intervals, and one sample was taken from a condensed phosphorite bed (7129/10-U-01, 66.5 m). The two cores are correlated with each other based on the lithology by Bugge et al. (49)

### Bulk Organic Properties.

Analyses of total organic carbon and total organic C-isotopes were performed at ISO-analytical (http://www.iso-analytical.co.uk). Samples were treated with HCl before analyses in order to remove carbonates. Replicate analyses on standards show SDs of <0.5‰.

### Extraction, Isolation, and Analysis of *n*-alkanes.

Between 5 and 30 g of dry sediment of each sample was extracted with a mixture of DCM/MeOH (7.5/1 vol/vol) using a Soxhlet device. Solvents containing the *n*-alkanes were then evaporated using rotary evaporation. The total lipid extracts were separated using small columns with activated aluminum-oxide. An aliphatic hydrocarbon fraction was eluted with hexane, a polar fraction with a mixture of DCM:MeOH (1:1 vol/vol). The hydrocarbon fraction was further cleaned using urea adduction, after which the adduct fraction (containing straight chain hydrocarbons) was dissolved in hexane for further analyses. Adduct fractions were injected on gas chromatography to test yield and to check for contamination. Mass spectrometry was used to identify compounds, and isotope ratio mass spectrometry was used for carbon isotope analyses at Utrecht University. For more details on the chromatography methods and alkane indices, refer to *SI Appendix*.

### Palynology.

Following standard palynological methods palynomorphs were extracted from powdered rock-samples using hydrochloric acid and hydrofluoric acid and sieving over a 7-μm mesh at University of Oslo. Only major groups of palynomorphs were distinguished, which were then grouped together into major vegetation types.

### Time Series Analysis.

Cyclostratigraphy of total gamma ray intensity at site 7128/12-U-01 (49) provides a floating astrochronology for the carbon isotope records. Time series analysis for the construction of an astronomical time scale follows typical procedures described in (102). The analysis was performed using a software *Acycle* version 2.2 (102) described fully in *SI Appendix*.

### Earth System Model Experiments.

In order to provide insight on the most plausible ^13^C-depleted carbon sources that disrupted the global carbon cycle and during the EPME, we use isotope inversion in cGENIE with our collected compound specific carbon isotope data. cGENIE was set up to the Late Permian boundary conditions (such as paleogeography and paleobathymetry; *SI Appendix*). This model has a one-dimensional atmosphere and a three-dimensional ocean with 16 vertical layers. cGENIE considers the following geochemical tracers in the atmosphere and oceans via ATCHEM and BIOGEM modules: O_2_, CO_2_, DIC, alkalinity, carbonate ion, stable carbon isotopes (^12^C and ^13^C), and nutrients (nitrate and phosphate).

Over >100,000-y timescales, inputs of carbon from silicate weathering on land and volcanic outgassing balance the carbonate burial output (103). Over shorter timescales (i.e., millennial to tens of thousands of years), exogenic carbon sources, such as methane hydrate and organic matter oxidation, can lead to imbalances in the carbon cycle, driving rapid changes in atmospheric *p*CO_2_. The biogeochemical model parameters and the rock weathering parameters are summarized in *SI Appendix*, Tables S1 and S2. The cGENIE model was run for 20,000 y in a closed system and another 200,000 y in an open system to allow for the balance between inputs from silicate weathering and degassing and outputs from carbonate burial. Afterward, seven experiments were set up using a range of plausible carbon sources (δ^13^C_source_ = −9, −15, −18, −25, and −30‰ that cover the range of δ^13^C values reported for CO_2_ from Koolau melt inclusions in ref. 69; we also include δ^13^C_source_ = −45‰ and −60‰ to cover scenarios associated with thermogenic and biogenic methane (104, 105) using the δ^13^C_algae_ records to derive surface ocean δ^13^C_DIC_ in the model inversion (refer to Fig. 5*F* for the evolution of surface ocean δ^13^C_DIC_ records and *SI Appendix* for the associated assumptions). The initial *p*CO_2_ was assumed to be ∼440 ppm based on stomatal ratio proxy using well-preserved cuticles from Southwest China (67), and the cGENIE model was tuned to an ice-free condition by adjusting the albedo. The models were run for 168,600 y to cover 251.941 to 251.772 Mya for short-chain, n-alkane–based inversion (from the onset of the CIE to 168.6 Kyr after the CIE onset; age 251.939 ± 0.031 Mya is marked in Fig. 5 as the age of the EPME based on ref. 106).

## Supplementary Material

Supplementary File

Supplementary File

Supplementary File

## Data Availability

All data presented in this study are available in the supporting information. The code necessary to run the cGENIE model is available at https://github.com/derpycode/cgenie.muffin, and all model outputs and instructions to replicate the model results are accessible at https://zenodo.org/record/4543684.
