# Supplementary material for: Massive and rapid predominantly volcanic CO2 emission during the end-Permian mass extinction
Source: Proc Natl Acad Sci U S A. 2021 Sep 7;118(37):e2014701118. doi: 10.1073/pnas.2014701118 (PMC8449420; doi:10.1073/pnas.2014701118)
Supplement: Supplementary File [file pnas.2014701118.sapp.pdf]

PNAS
www.pnas.org

Supplementary Information for

Massive and rapid predominantly volcanic CO_2 emission during the end-Permian mass extinction.

Ying Cui[1,*], Mingsong Li[2,*], Elsbeth E. van Soelen[3], Francien Peterse[4], Wolfram M. Kürschner[3,*]

[1]Department of Earth and Environmental Studies, Montclair State University, Montclair, NJ 07043, USA

[2]School of Earth and Space Sciences, Peking University, Beijing 100871, China

[3]Department of Geosciences, University of Oslo, Oslo, Sem Sælands vei 1, 0371, Norway.

[4]Department of Earth Sciences, Utrecht University, Princetonlaan 8A, 3584 CB Utrecht, Netherlands.

*Corresponding author: Wolfram M. Kürschner, Ying Cui and Mingsong Li

Email: w.m.kurschner@geo.uio.no; cuiy@montclair.edu; msli@pku.edu.cn

This PDF file includes:

Supplementary text
Figures S1 to S26
Tables S1 to S8
SI References

Supporting information Text

S1. Short description the location and core material

The cores 7128/12 – U- 01 and 7129/10-U-01 are two parallel cores drilled by the IKU (now SINTEF) Petroleum Research on the eastern part of the Finnmark platform (Fig. S1) in the southeastern part of the Barents Shelf offshore northern Norway. This geological structure represents a carbonate platform with underlying fault blocks containing a succession of marine mixed siliciclastic sediments and carbonates of Carboniferous – Triassic age.

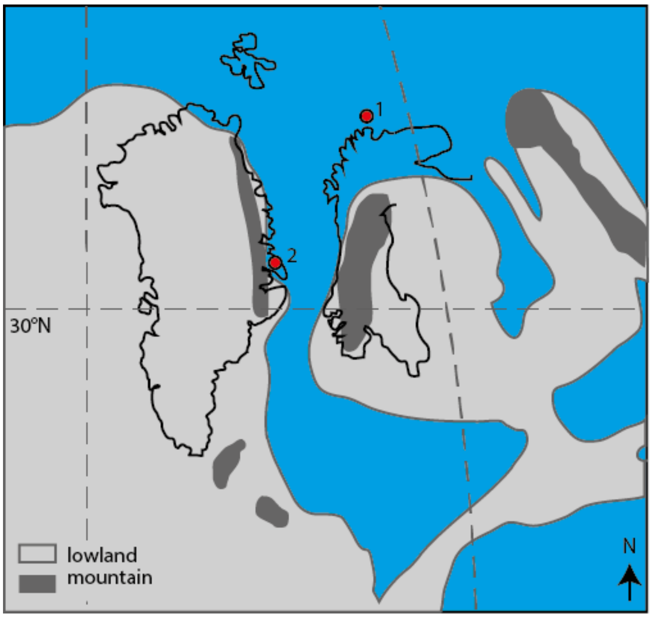

Figure S1 Paleogeographical map of the Late Permian, with former and current coastlines. Indicated are 1) the location of Finnmark cores 7128/12-U-01 and 7129/10-U-01, and 2) the Kap Stosch section on East-Greenland discussed in the main text. Map is adapted from (1).

The sedimentology, lithostratigraphy and biostratigraphic correlations of the cores have been described extensively in several papers (2-5).

Core photos and descriptions are available at https://www.sintef.no/projectweb/ik-stratigraphic-drilling/

19 samples (Fig. S2) were analyzed for this study from the two parallel cores 7128/12-U-01 and 7129/10-U-01 in stratigraphic order. These samples cover continuously the Permian-Triassic boundary interval from the Upper Permian Røye Formation of the Tempelfjorden Group and the lowermost Triassic Havert Formation of the Sassendalen Group.

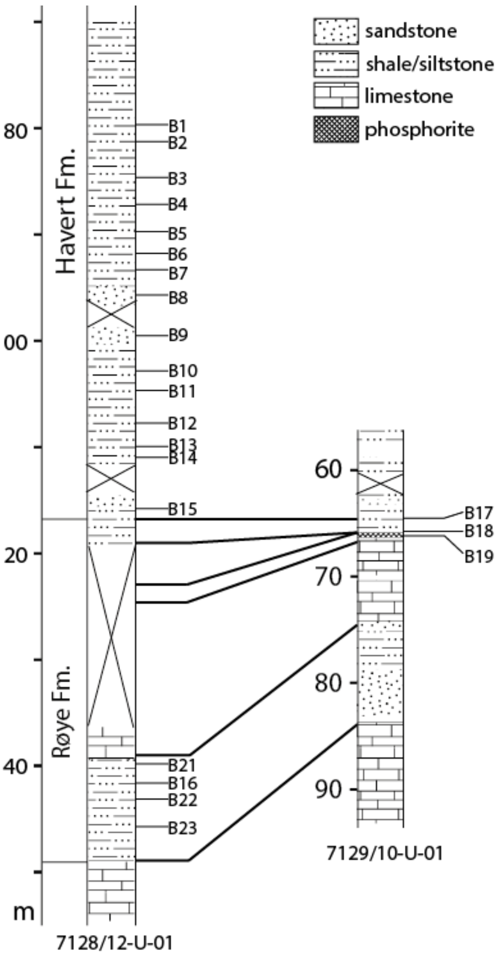

Figure S2. Schematic lithology and correlation of the two parallel cores 7128/12-U-01 and 7129/10-U-01 after (2).

S2. Additional organic geochemical information

S.2.1 Gas chromatography

Samples were injected on-column, with helium as carrier gas set at constant pressure (100 kPa). The GC was fitted with a CP-Sil 5CB fused silica capillary column (30 m 0.32 mm i.d.) and a flame ionization detector (FID). The oven program started at 70 °C, then heated up to 130 °C at a rate of 20 °C min^{-1}, and then continued with 4 °C min^{-1} up to 320 °C after which the temperature was kept stable for 20 min. A standard containing known straight chain *n*-alkanes was used to identify the *n*-alkanes in the samples. Also, mass spectrometry (GC–MS) was used on one sample to confirm the identification on the straight chain *n*-alkanes. The carbon preference index (CPI) and average chain length (ACL) were based on the relative abundance of the long chain *n*-alkanes, according to the following equations.

$$\text{CPI} = = 0.5 * (\frac{(C25+C27+C29+C31+C33)}{(C24+C26+C28+C30+C32)} + \frac{(C25+C27+C29+C31+C33)}{(C26+C28+C30+C32+C34)}$$

$$\text{ACL} = \frac{(25*[C25]+27*[C27]+29*[C29]+31*[C31]+33*[C33])}{([C25]+[C27]+[C29]+[C31]+[C33])}$$

S.2.2 Gas chromatography – isotope ratio mass spectrometry.

The same type and size of column and similar oven program are used as for GC, with the exception of the He flow, which was kept on constant flow (instead of constant pressure). Every day, the performance of the instrument was tested and standards were measured. For most samples, duplicates were measured (exception of sample B7 analyzed 4x) with the results averaged to obtain a mean value and standard deviation. The reported average values and the standard deviation are based on the C-isotopes are reported in ‰ relative to Vienna Pee Dee Belemnite (VPDB).

All *n*-alkane data and compound specific isotope values are summarized in Tables S7 and S8.

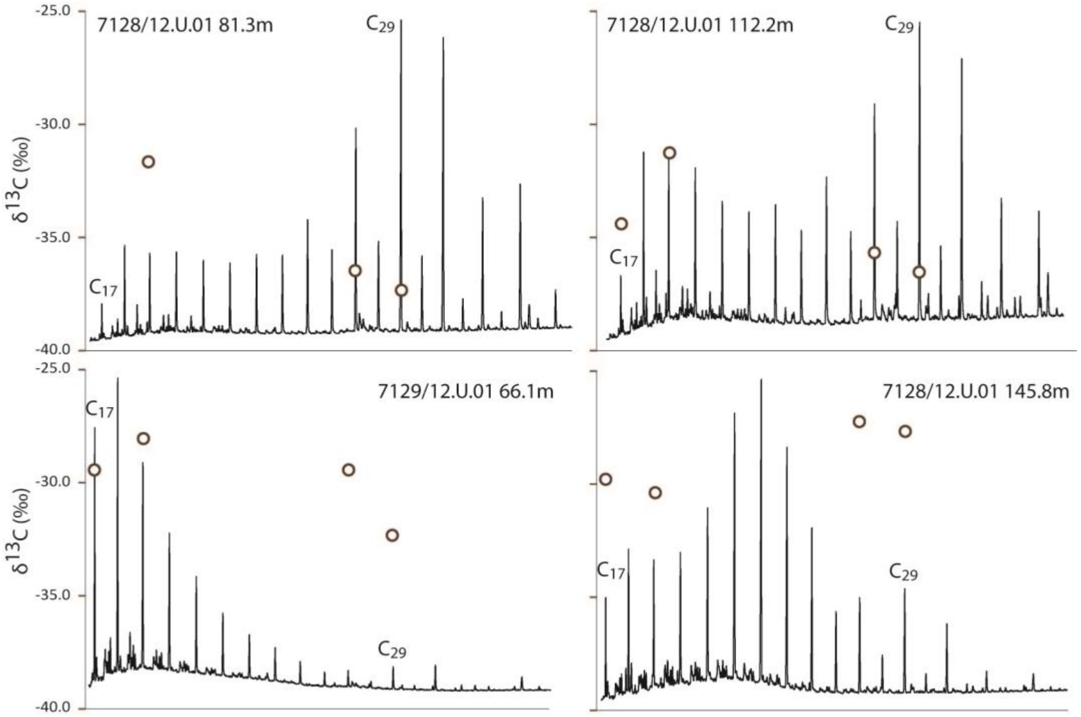

Figure S3. Gas chromatogram traces of saturated hydrocarbon fractions (after urea adduction) of Finnmark core 7128/12-U-01 and 7129/10-U-01. $\delta^{13}C$ values of selected alkanes (C17, C19, C27 and C29) are indicated.

S2.3 Carbon isotope records and vegetation changes

The extent to which local terrestrial ecosystem reorganization may have modified the $\delta^{13}C_{org}$ can be assessed by evaluating the differences between $\delta^{13}C_{wax}$ and $\delta^{13}C_{algae}$. The strong correlation between $\delta^{13}C_{org}$ and $\delta^{13}C_{C27}$ (r^2=0.90) and $\delta^{13}C_{C29}$ (r^2=0.82) (Fig. S4A) confirms the previous suggestion that the organic matter at the Finnmark site is primarily of terrestrial origin with little input of marine organic matter (6). There is a strong correlation between $\delta^{13}C_{org}$ and $\delta^{13}C_{C27}$ (r^2=0.90) and $\delta^{13}C_{C29}$ (r^2=0.82) (Fig. S4A) which indicates that the bulk $\delta^{13}C_{org}$ is mainly influenced by terrestrial organic matter. Moreover, there is a negative correlation between $\delta^{13}C_{org}$ and both relative and absolute abundances of pteridosperm pollen for the interval above the first carbon isotope excursion (Fig. S4B).

S2.4 *Effects of vegetation changes and hydrological cycle on leaf wax carbon isotope excursion*

The first and second negative excursions in the $\delta^{13}C_{wax}$ coincide with increases in ACL and pollen/spore counts (Fig. 2), suggesting that changes in plant communities may have driven the $\delta^{13}C_{wax}$ towards lower values. This is supported by negative correlations between $\delta^{13}C_{org}$ (also $\delta^{13}C_{wax}$) and abundances of pteridosperm pollen (both relative and absolute) for the interval above the first carbon isotope excursion (Fig. S4B and Fig. S5). The pollen record indicates pteridosperm (seed ferns) dominated woody vegetation in the lower part of core, gradually transitioning into more spore producers (e.g., lycopsids, ferns, mosses) around 116 m core depth. Subsequently, conifers increase between 103 and 95 m, followed by a short-lived return of spore producers (92-89 m), after which pteridosperms once more become dominant. The gradual increase in $\delta^{13}C_{wax}$ between 105 and 95 m (Fig. 2F, G) coincides with a temporary increase in the relative abundance of conifer pollen (Fig. 2A). The amplification of the carbon isotope excursion (CIE) magnitude in $\delta^{13}C_{wax}$ due to changes in vegetation type is supported by recent growth chamber experiment by (7). Significant differences in carbon isotope discrimination between extant spore plants versus seed plants has been observed by Porter et al. (7), in which spore-producing plants show higher carbon isotope fractionation ($\Delta^{13}C$ = ~22‰ for ferns, where $\Delta^{13}C \approx \delta^{13}C_{atm} - \delta^{13}C_{plant}$) compared to conifers ($\Delta^{13}C$ = ~18‰ for gymnosperm) at the same level of environmental O_2:CO_2 condition (8). The carbon isotope fractionation of the pteridosperms, an extinct group of seed plants is unknown, but Late Permian seed ferns comprise different genera of peltasperms that co-occurred with conifers in warm semi-arid climate conditions (9). Given that both seed plant groups had similar environmental preferences, we presume that peltasperms exhibit a similar carbon isotope discrimination as conifers. This presumption appears to be consistent with the observed $\delta^{13}C_{org}$ pattern given that pre-CIE interval is composed of 80% seed-producing plants (13% conifers and 64% pteridosperms) and 18%

spore-producing plants with an average $\delta^{13}C_{plant}$ of -24‰. Based on these observations, we infer a decrease from 80% to 22% seed-producing plants and an increase from 18% to 75% spore-producing plants alone during the peak CIE would have lowered the $\delta^{13}C_{wax}$ by ~2‰ (*SI Appendix* Fig. S5). Although the effect of higher temperatures on fractionation during photosynthesis is considered to be relatively small (10), changes in precipitation have known effect on carbon isotope fractionation (10, 11). Unfortunately, the paleohydrological pattern of the study site is not well constraint, and we are not able to quantify the precipitation effect on the CIE magnitude. However, there is known CO_2 effect on carbon isotope fractionation in the fossil records (10, 12, 13), which allows us to quantify the amplification of the leaf wax CIE based on our best-fit model results. Rising atmospheric CO_2 levels, a likely consequence of the large-scale volcanic activity in the late Permian (14, 15), can result in increased carbon isotope fractionation (12, 16, 17) and amplify the negative CIE in $\delta^{13}C_{wax}$ records by ~6‰. The pCO_2 effect is quantified using the following equation from Schubert and Jahren (12):

$$\Delta^{13}C = [(28.26)(0.21)(pCO_2+25)]/[28.26+(0.21) \times (pCO_2 + 25)]$$

The following table shows the effect on the carbon isotope fractionation from increase in pCO_2 based on our best-fit scenario associated with an average $\delta^{13}C$ of -15‰ for the CO_2 source:

$\Delta_{initial}$	Δ_{peak} (-15‰)	ΔpCO_2	pCO_2 effect
21.9	27.8	7391	5.9

If we allow the initial pCO_2 to be higher (2800 ppm) following Cui et al. (14), the following table shows the effect on the carbon isotope fractionation for pCO_2 increase to 10,191 ppm on our best-fit scenario associated with an average $\delta^{13}C$ of -15‰ for the CO_2 source:

$\Delta_{initial}$	Δ_{peak} (-15‰)	ΔpCO_2	pCO_2 effect
27.0	27.9	7391	0.9

Adding together, changes in regional vegetation type, variations in precipitation pattern and rising atmospheric CO_2 levels may have amplified the magnitude of the CIE recorded in

$\delta^{13}C_{wax}$ in the Finnmark Platform by as much 6‰, leaving the ~5‰ CIE similar to the global CIE records (Fig. 3D-F). Note that we also offer an alternative interpretation of the larger CIE in $\delta^{13}C_{wax}$ due to a disequilibrium between the surface ocean and the atmosphere under the condition that there is only a small amplification in the CIE due to the pCO_2 effect on carbon isotope fractionation of C_3 land plants if an initially larger pCO_2 is assumed prior to the mass extinction.

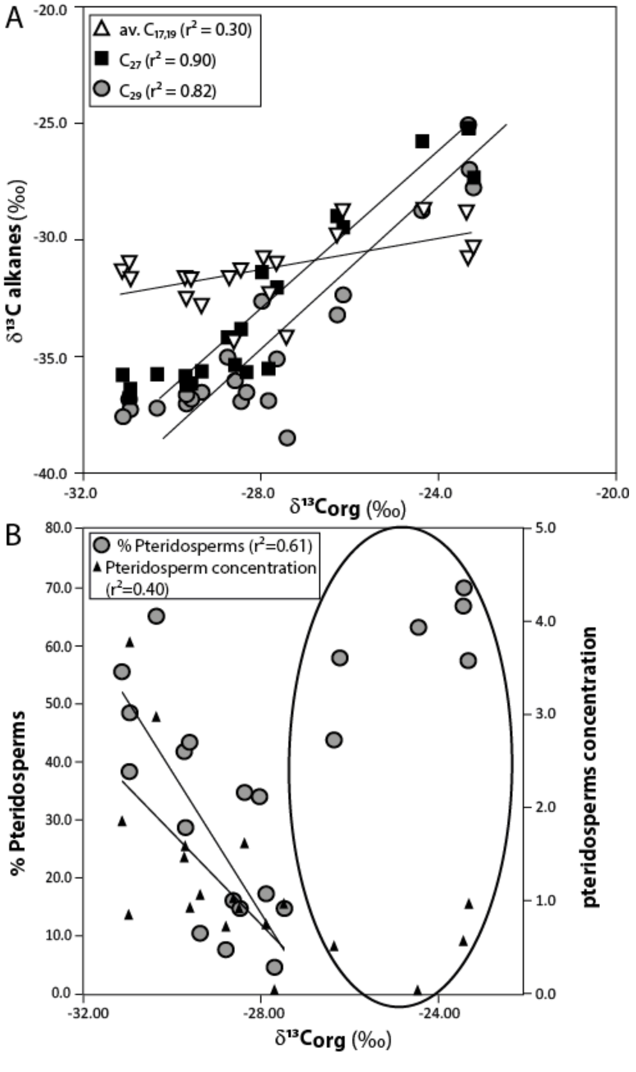

Figure S4. A) Correlations between bulk organic $\delta^{13}C$ values and compound specific $\delta^{13}C$ values of cores 7128/12-U-01 and 7129/10-U-01, B) correlations between bulk $\delta^{13}C$ data and the relative and absolute abundance of Pteridosperm pollen from cores 7128/12-U-01 and 7129/10-U-01. The data that does not fit the correlation (within the circle) comes from below the first isotope shift (>120m in core 7128/12-U-01, or >70 m in core 7129/10-U-01).

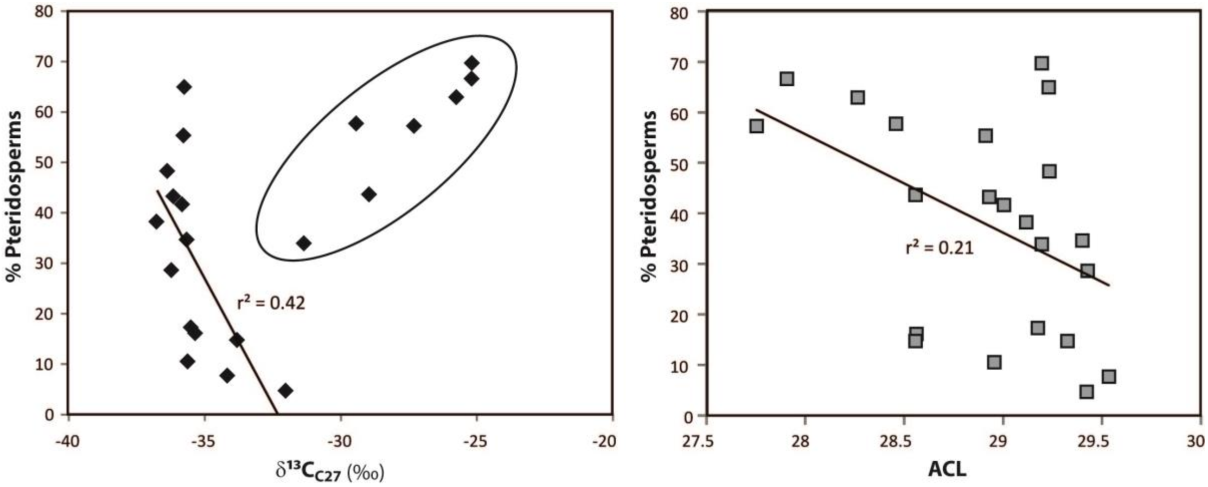

Figure S5 Correlations between carbon isotope composition of the C_{27} alkane and relative abundance of Pteridosperm pollen (left) and the average chain length (ACL) and relative abundance of Pteridosperm pollen (right). In the left panel: the data points within the circle are from below the first negative shift and were not included in the regression. NOTE: There is no correlation between spore- or conifer-concentrations and $\delta^{13}C_{org}$ or $\delta^{13}C_{wax}$. Spore-plants can fractionate stronger than seed-plants (8) and changes in spore-plant biomass is thus expected to affect the carbon isotope signal. The lack of correlation might be explained by the lower abundance of spores compared to pteridosperms, or the fractionation was different in these extinct plant species.

S3. Time-series analysis

S3.1 Introduction

The sedimentary record of Milankovitch cycles of orbital eccentricity, tilt, and precession can be used to generate a high-resolution astronomical time scale for the carbon cycle perturbations across the Permian-Triassic boundary (18, 19). The high sedimentation rate of the siliciclastic sediments at studied site offers high-resolution records to establish an astronomical time scale based on cyclostratigraphy, which enables a high-resolution correlation with the carbon isotope record at Meishan section of South China.

S3.2 Materials and methods

Time series analysis for the construction of an astronomical time scale follows typical procedures described in (20). We used a software Acycle v1.0 (20) (https://github.com/mingsongli/acycle) to accomplish the following tasks:

1. A high-resolution gamma ray profile of at site 7128/12-U-01 (2) was digitalized using "Plot Digitizer" tool in Acycle.

2. Digitalized gamma ray data (Fig. S6) is irregularly spaced. This necessitates interpolation to generate uniformly spaced depth series because many following analyses such as multi-taper power spectral analysis, filtering and red noise models require uniformly spaced data series. The gamma ray data was interpolated using a 0.1-m sampling rate.

3. Many time series analysis techniques assume the data have a zero-mean and a Gaussian distribution. The gamma ray value of 564 API at 124.2 m is much high than other values. This outlier can cause distortion of the power spectrum (21). So, it was eliminated by manually changing the value to 200 (Fig. S7). Then the gamma ray profile was detrended using a 50-m local regression smoothing (LOWESS) method (22) to remove the long-term trend (Fig. S7).

4. The detrended gamma ray profile was analyzed using both periodogram and 2π multi-taper method power estimator (23) and a least squares analytic fit of the first-order autoregressive AR(1) model to a median-smoothed spectrum estimate, which is also known as the robust AR(1) model (24). These were to obtain significant periodicities in the gamma ray series (Fig. S8).

5. Variations of periodicities across the gamma ray profile can be shown in the evolutionary power spectra (25), which is estimated using "Evolutionary Spectral Analysis" tool in Acycle (Fig. S9).

6. To unravel the value of the optimal sedimentation rate, two statistical methods, i.e., Correlation Coefficient (COCO) (26) and the time scale optimization (TimeOpt) (27) were used. For the COCO method, the power spectra of gamma ray series are compared to the astronomical solution power spectrum for a given sedimentation rate using the concept of a correlation coefficient. The highest values of the correlation coefficient are evaluated, and their significance are estimated by comparison to values expected from a chance correlation using Monte Carlo methods. The sedimentation rate with the highest value of correlation coefficient is considered to be the optimal sedimentation rate. The age of the Permian-Triassic boundary is 251.9 Ma (28), which is beyond the 249 Ma age limit of the Laskar solutions (29, 30). Therefore, we use the extended Berger89 solution (31), which gives seven astronomical cycles of 413 kyr, 123 kyr, 95 kyr, 44.92 kyr, 33.53 kyr, 21.17 kyr, and 17.72 kyr, for the COCO evaluation. Test sedimentation rates range from 2 to 30 cm/kyr with a step of 0.1 cm/kyr. The number of Monte Carlo simulations is 5000.

The second TimeOpt method simultaneously optimizes the precession-eccentricity variances and eccentricity-related amplitude modulation of the precession frequency band by searching for a range of test sedimentation rate. For each test sedimentation rate, the proxy series is converted to time domain. Then the series is linearly regressed on a model series with precession and eccentricity frequencies, where the model series is determined by least square estimation. The performance of the linear regression is measured as the correlation coefficient r^2_{power}. Meanwhile, the series in time domain is Taner band-pass filtered and Hilbert transformed to extract the precession related amplitude envelope (32). The envelope is linearly regressed on a model series with eccentricity cyclicities. The result of this linear regression is reported as $r^2_{envelope}$. A combination of these information can be indicated by $r^2_{opt} = r^2_{power} * r^2_{envelope}$. Therefore, the highest value of r^2_{opt} signs the corresponding sedimentation rate is probably the optimal sedimentation rate. The significant intervals of these values of r^2_{power}, $r^2_{envelope}$ and r^2_{opt} are evaluated using Monte Carlo generated red-noise series using the estimated AR(1) value of the gamma ray data. Here 200 test sedimentation rates range from 2 to 30 cm/kyr. The eccentricity and

precession cycles in La2004 solution are 409.6 kyr, 132.13 kyr, 124.12 kyr, 98.70 kyr, 95.26 kyr, 20.69 kyr, 19.69 kyr, 17.13 kyr, and 17.00 kyr (29). This solution enables more precession cycles in the TimeOpt evaluation than the Berger89 solution (31). We use 2000 Monte Carlo simulations to evaluate the significance level of the r^2 values. Taner bandpass cut-off frequencies are 0.0394 and 0.0732 cycles/kyr with a roll-off rate of 10^{12}.

7. Using the detected optimal sedimentation rate in Step 6, the gamma ray series was linearly converted to time domain, which provides a floating astronomical time scale. We set the initial CIE to be 0 kyr for the construction of the final astronomical time scale.
8. We used the Gauss band-pass filter (25) to identify the potential astronomical cycles using Acycle's "Filtering" toolbox.

S3.3 Results

Time series analysis of total gamma ray intensity at site 7128/12-U-01 (2) provides a floating astronomical time scale for the carbon isotope records. Power spectrum of the gamma ray series shows that the dominated cycles are 23 m, 7.4 m, 5.5 m, 4 m, and 2.7 m (Fig. S8). Evolutionary power spectrum indicates the 23 m cycles represent the dominated cyclicity throughout the series; only in the Permian-Triassic boundary interval (ca. 125-105 m) the leading cyclicities are 5-7 m cycles (Fig. S10). The optimal sedimentation rate is ~22.4 cm/kyr based on both results of COCO and TimeOpt methods. The COCO plot shows the highest peak of sedimentation rate is 20.2 cm/kyr, with two slight lower peaks at 4.8 cm/kyr and 6.2 cm/kyr (Fig. S11). All of these three peaks have lower than 1% null hypothesis significance levels, i.e., all have high confidence levels. TimeOpt analysis supports that the optimal sedimentation rate is 22.4 cm/kyr. $r^2_{envelope}$ plot shows the highest peak of $r^2_{envelope}$ at 22.4 cm/kyr. In comparison, there are many peaks in the r^2_{power} plot, and a relative flat top in the r^2_{opt} plot ranging from 23 to 28 cm/kyr. However, the null hypothesis analysis of these r^2 values shows that only the $r^2_{envelope}$ has a significance level lower than 1% (Figs. S12, S13). Taken together, the mean sedimentation rate of the gamma ray series should be at 22.4 cm/kyr. This sedimentation rate enables a 432 kyr floating astronomical time scale for the carbon isotope records. The interval between the two carbon isotope excursions (CIEs) has a ~110 kyr (105-115 kyr) duration based on the astrochronology that assumes a stable sedimentation rate. In comparison bandpass filtering suggests this interval contains ~4

precession cycles, i.e., ~80 kyr in duration assuming the precession cycle was 20 kyr in 251.9 Ma (Fig. S14).

S3.4 Discussion

The CIE duration encompasses ~110 kyr, which is comparable with the Meishan section of South China, the Global Boundary Stratotype Section and Point (GSSP) for the basal Triassic. Cyclostratigraphy of the Meishan uranium series suggests the two pulses of extinction span about 40 kyr and the largest $\delta^{13}C_{carb}$ anomaly lasted about 6 kyr (19). Other estimations of the end-Permian extinction interval at Meishan are 60 ± 48 kyr based on radioisotope dating (28) and 83 kyr based on cyclostratigraphy (18). However, the Meishan section is condensed, which limits the confidence of the astrochronology. Here the continuous Finnmark section with very high sedimentation rate enables a reliable estimation of the CIE duration of ~110 kyr. At both Finnmark and Meishan sections the end-Permian $\delta^{13}C$ excursion started at the peak of one 100 kyr cycle (Fig. S14), providing additional evidence for the global correlation of the carbon isotope record.

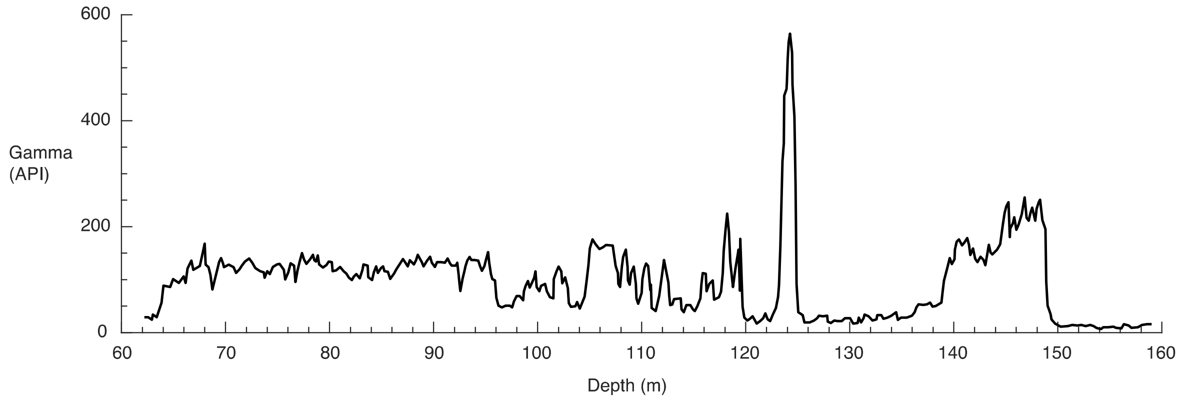

Figure S6. Digitalized gamma ray series at site 7128/12-U-01 (2).

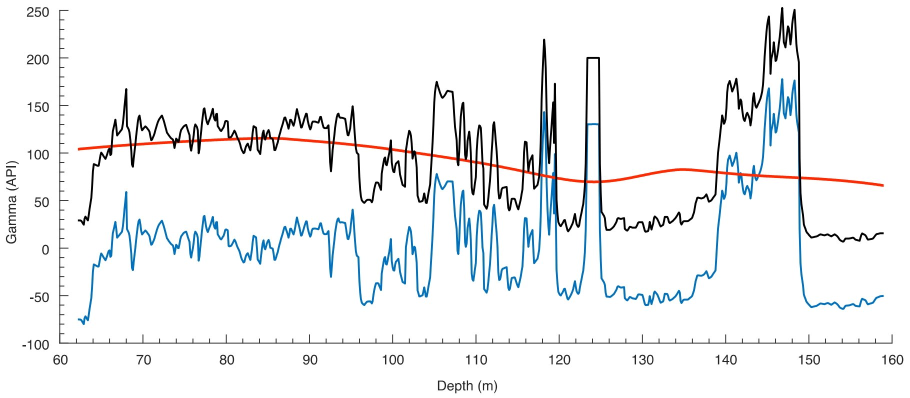

Figure S7. Gamma ray series removing outliers at ~124.2 m (thin black) is shown with a 50 m LOWESS long term trend (thick red) and detrended gamma ray data (thin blue).

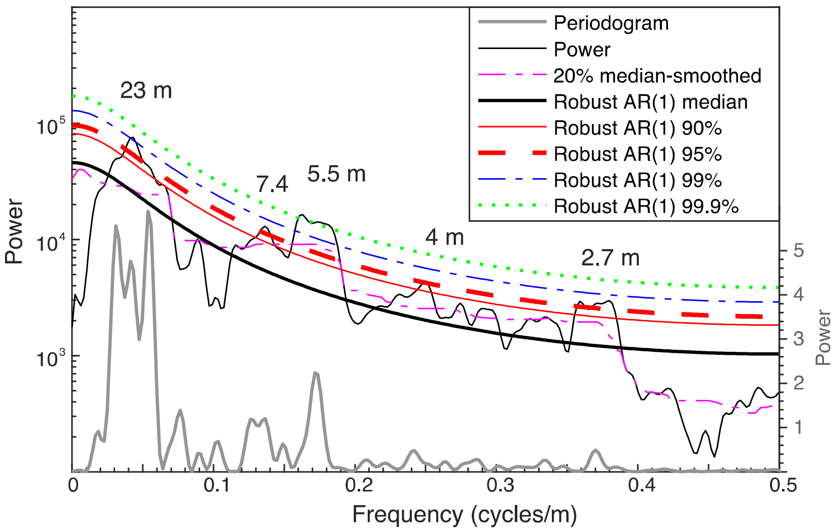

Figure S8. 2π multi-taper method power spectrum (thin black) and periodogram (thick gray) of the gamma ray series shown with robust red-noise models. The red-noise fit to the spectrum is based on the best fit (thick black) to the log power of the 20% median-smoothed spectrum (dashed pink). The 90% (solid red), 95% (dashed red), 99% (blue dashed line), and 99.9% (dot green) confidence limits are also shown. Cycle wavelengths are also marked.

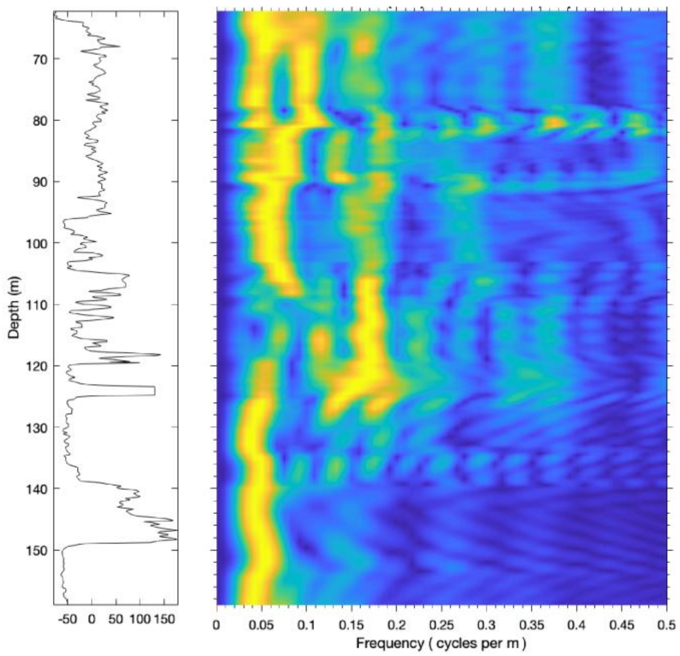

Figure S9. Gamma ray series shown with evolutionary fast Fourier transform spectra (25). The sliding window is 30 m with a sliding step of 0.1336 m. Both ends of the gamma ray series is zero-padded to generate an equal-depth evolutionary spectra.

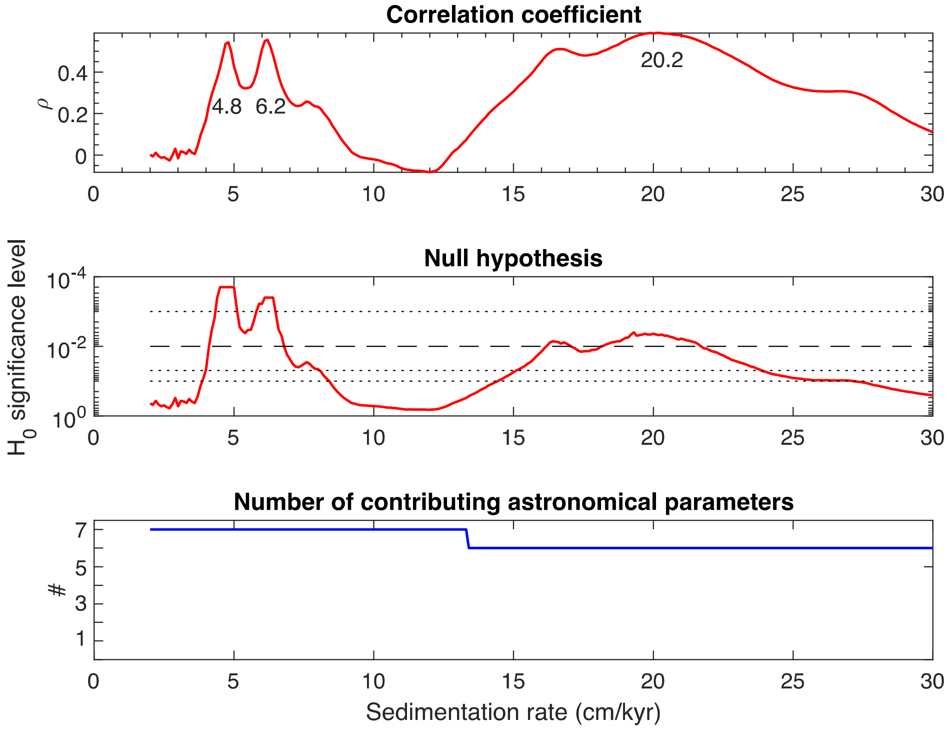

Figure S10. COCO generated sedimentation rate. (a) The COCO analysis shows the highest peak at 20.2 cm/kyr with two lower peaks at 4.8 cm/kyr and 6.2 cm/kyr. (b) Null hypothesis testing of the data series indicates that all three deposition rates have significance levels less than 1%. (c) Number of contributing astronomical parameters.

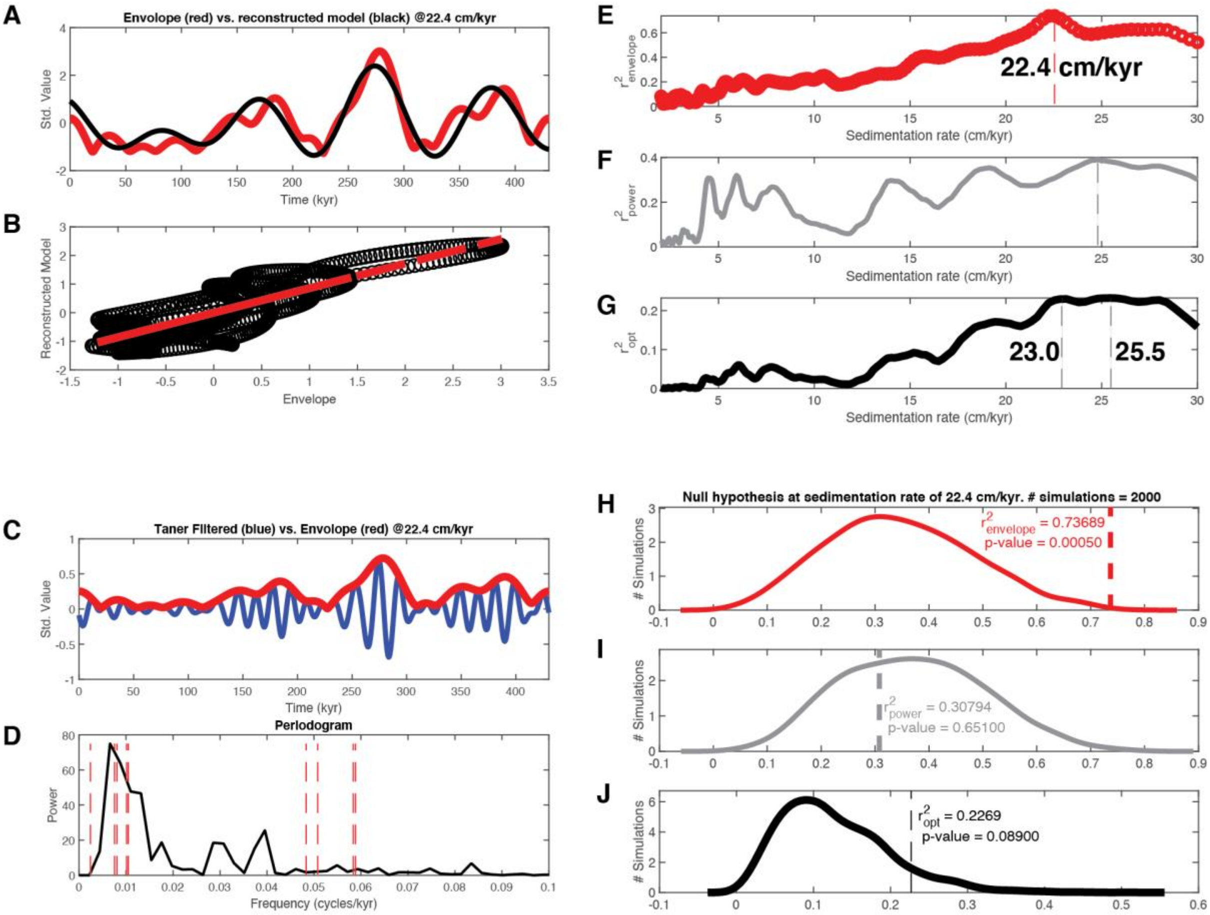

Figure S11. TimeOpt generated sedimentation rate. (A) Envelope of filtered precession signals (red) and reconstructed eccentricity model (black) at sedimentation rate of 22.4 cm/kyr. (B) Correlation of the envelope of filtered precession signals and reconstructed eccentricity model. (C) Taner filtered time series using a sedimentation rate of 22.4 cm/kyr (blue) and its envelope (red). (D) Periodogram of the gamma ray time series using a sedimentation rate of 22.4 cm/kyr and input astronomical frequencies. (E) Squared correlation coefficient for the amplitude envelope fit ($r^2_{envelope}$) and (F) the spectral power fit (r^2_{power}). (G) Combined envelope and spectral power fit (r^2_{opt}) at test deposition rate. (H-J) Summary of 2000 Monte Carlo simulations with AR(1) surrogates used to evaluate the significance levels of the maximum observed $r^2_{envelope}$ (H), r^2_{power} (I), and r^2_{opt} (J).

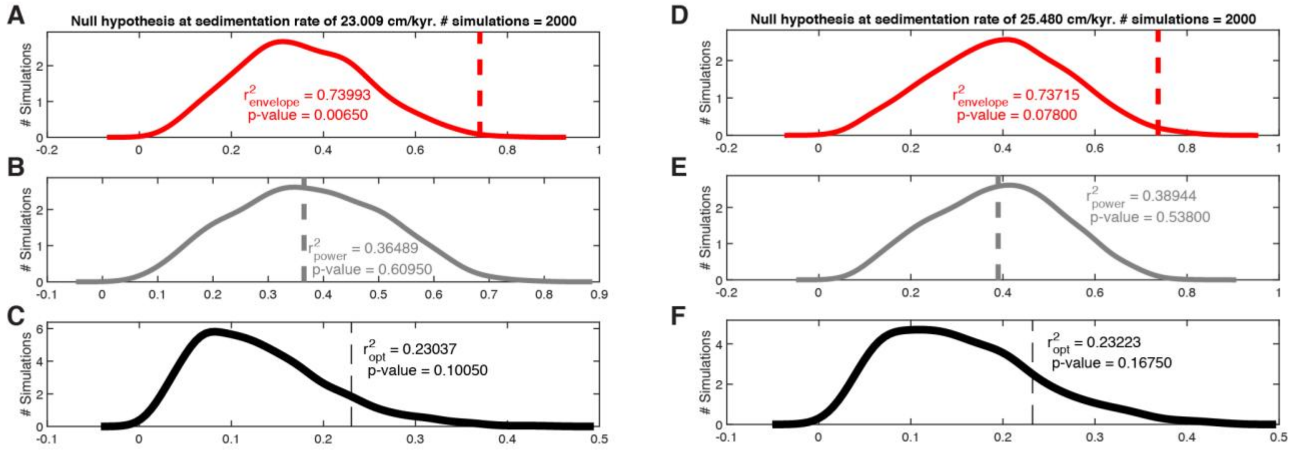

Figure S12. Summary of 2000 Monte Carlo simulations with AR(1) surrogates used to evaluate the significance levels of the maximum observed $r^2_{envelope}$ (A,D), r^2_{power} (B,E), and r^2_{opt} (C,F) at sedimentation rate of 23.0 cm/kyr (a-c) and 25.48 cm/kyr (d-f).

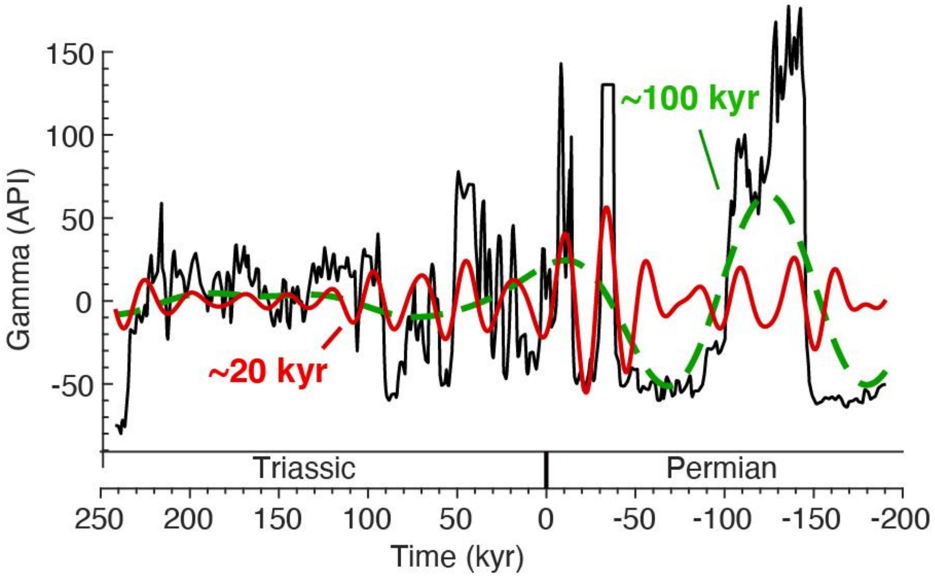

Figure S13. Time calibrated gamma ray series (black) is shown with its ~20 kyr (red) and ~100 kyr (dashed green) Gauss bandpass-filtered cycles (passband: 0.045 ± 0.01 and 0.0085 ± 0.0025 cycles/kyr, respectively). The onset of the Permian-Triassic carbon isotope excursion is set to 0 kyr.

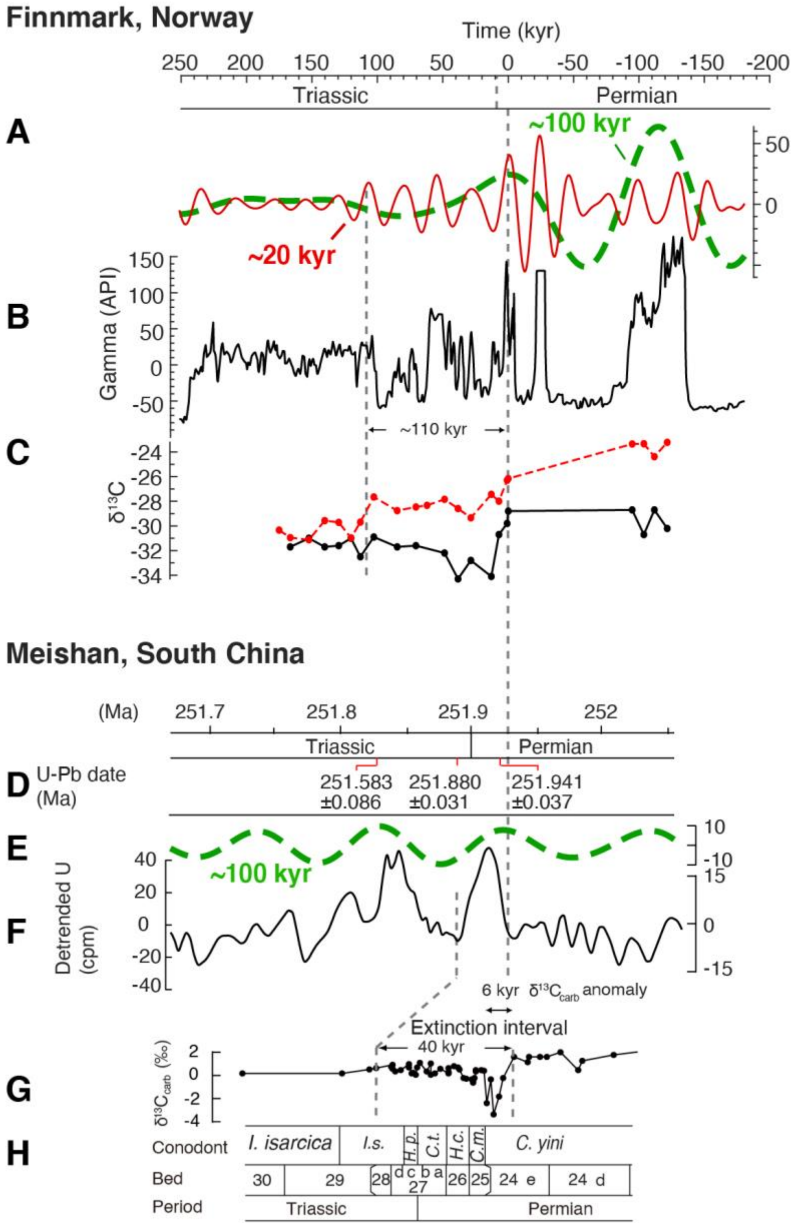

Figure S14. Cyclostratigraphic correlation of the Finnmark section of Norway (A-C) and the Meishan section of South China (D-H). (A-B) Tuned gamma and its ~100 kyr and ~20 kyr filter outputs are from Fig. S8. (C) Tuned carbon isotope $\delta^{13}C$ of average of C_{17} and C_{19} alkane (solid black) and $\delta^{13}C_{org}$ (dashed red). (D) Radio-isotope dating of volcanic ash beds at Meishan (28). (E) Filtered 100 kyr cycles of tuned uranium series (F) at Meishan are from ref. (19) and are shown with $\delta^{13}C_{carb}$ data (G) (33), conodont zones, and bed numbers (H) (34).

S4. cGENIE model setup and code availability

The code of cGENIE (carbon cycle Grid Enabled Integrated Earth system model) used to run the model experiment can be found on Github at: https://gibhub.com/derpycode/cgenie.muffin. User and base configuration files for the experiments shown in this paper and detailed experiment descriptions and the command line to run experiment can be found at: https://zenodo.org/record/4543684, from which all of the model results are available for download and analysis.

The cGENIE model used in this work is classified as an Earth system model of intermediate complexity (EMIC). It is composed of a 2D atmospheric chemistry module, a 3D ocean biogeochemistry module, a 3D sediment geochemistry module, and a 2D rock weathering geochemistry module coupled to an energy-moisture balance module, dynamic-thermodynamic sea-ice module and a 3D ocean circulation module through transferring materials via an interface (Fig. S15). The focus of the experiment set up is the carbon cycle model as described in Colbourn et al. (35). Spatially resolved carbonate and silicate weathering processes and deposition of sediments in the ocean are both included. In order to simulate the carbon emission, a two-stage model spin-up is run, followed by a carbon isotope inversion using the $\delta^{13}C$ of the dissolved inorganic carbon (DIC) as the primary forcing. The paleogeography has 36 × 36 land grid cells and 16 depth levels in the ocean (Fig. S16). The physical and biogeochemical parameters are listed in Table S1 according to (36-41). Selected model outputs are shown in Figures S17-S26 and model parameterizations and results summary are shown in Tables S2-S4. Tables S5 and S6 show the summary of carbon emission estimates and the carbon isotopic values of the CO_2 related to the Siberian Traps volcanism.

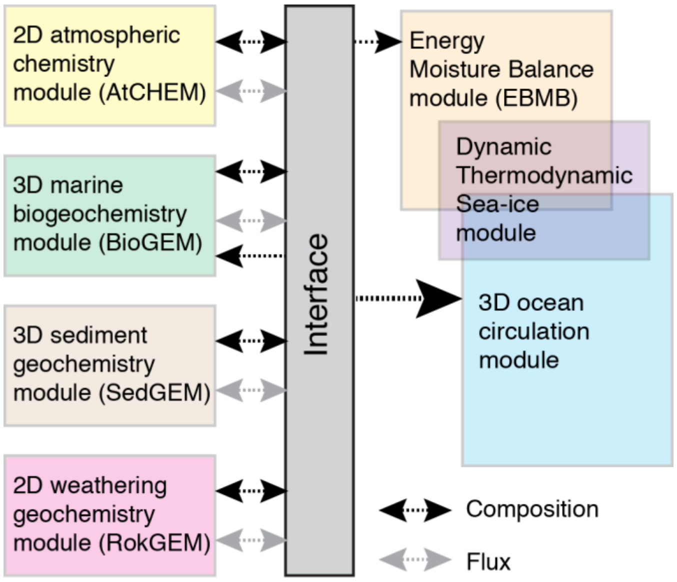

Figure S15. cGENIE schematics of the seven modules used in the Permian cGENIE model and their interactions. Black arrows represent compositional information, and grey arrows represent fluxes.

Table S1. Biogeochemical parameters for the Permian cGENIE used in this study.

Parameter	Description	Value	Units	References
$u_0^{PO_4}$	Maximum PO_4 uptake (removal) rate	8.99	µmol kg^{-1} yr^{-1}	(14)
K^{PO_4}	PO_4 Michaelis-Menton half-saturation concentration	0.89	µmol kg^{-1}	Same as above
r^{POC}	Partitioning of POC export into fraction #2	0.0557	-	Same as above
l^{POC}	*e*-folding depth of POC fraction #1	590	m	Same as above
l_2^{POC}	*e*-folding depth of POC fraction #2	$1e^6$	m	Same as above
$r_0^{CaCO_3:POC}$	$CaCO_3$:POC export ratio scalar (set to no pelagic carbonate production)	0	-	Same as above
l^{CaCO_3}	*e*-folding depth of $CaCO_3$ fraction #1	1890.5	m	Same as above
$l_2^{CaCO_3}$	*e*-folding depth of $CaCO_3$ fraction #2	$1e^6$	m	Same as above

Table S2. Key climate-relevant parameters at the end of the 2nd stage spinup (at equilibrium).

Parameter	Description	Value	Units	References
$T_{average\ land}$	Global average land surface temperature	12.8	Degree C	This study
T_{max}	Maximum land surface temperature	30.2	Degree C	This study
T_{min}	Minimum land surface temperature	-28.1	Degree C	This study
$T_{average\ air}$	Global average air temperature	19.3	Degree C	This study
$T_{average\ ocean}$	Global average ocean temperature	5.4	Degree C	This study
$T_{surface\ ocean}$	Global surface ocean temperature	22.0	Degree C	This study
$T_{deep\ ocean}$	Global surface ocean temperature	3.0	Degree C	This study
$R_{average}$	Global average land surface runoff	254.9	mm yr^{-1}	This study
R_{max}	Maximum land surface runoff	1531.4	mm yr^{-1}	This study
R_{min}	Minimum land surface runoff	0	mm yr^{-1}	This study
pCO$_2$	Global average atmospheric pCO$_2$	440	ppm	(42)
F_{ALK}	Alkalinity weathering flux	53.3	Tmol yr^{-1}	This study
F_{DIC}	Dissolved inorganic carbon weathering flux	26.7	Tmol yr^{-1}	This study
F_{Ca}	Calcium weathering flux	26.6	Tmol yr^{-1}	This study
$\delta^{13}C_{weathering}$	$\delta^{13}C$ of dissolved inorganic carbon from weathering flux	-0.5	‰	This study
$\delta^{13}C_{atm}$	$\delta^{13}C$ of atmospheric CO_2	-5.3	‰	This study
$\delta^{13}C_{DIC}$	$\delta^{13}C$ of global dissolved inorganic carbon	1.1	‰	This study
$\delta^{13}C_{benthic\ DIC}$	$\delta^{13}C$ of benthic dissolved inorganic carbon	0.9	‰	This study
$\delta^{13}C_{surface\ DIC}$	$\delta^{13}C$ of surface ocean dissolved inorganic carbon	2.4	‰	This study

$\delta^{13}C_{sediment}$	$\delta^{13}C$ of reef sediments	3.1	‰	This study

Table S3. Model assumptions and summary of key model results.

**Experiment ID suffix**	**Source $\delta^{13}C$ (‰)**	**Peak Emissions (Gt C yr^{-1})**	**Peak Cumulative Emissions (Gt C)**	**ΔSST (^oC)**	**ΔpCO_2 (ppmv)**	**ΔpH**
*shortchain_9	-9	22.2	97508	18.3	30219	-1.6
***shortchain_15**	**-15**	**4.5**	**36212**	**11.6**	**7391**	**-1.1**
*shortchain_18	-18	3.0	28021	10.2	5220	-0.9
*shortchain_25	-25	1.7	18475	8.2	3116	-0.8
*shortchain_30	-30	1.5	14883	7.3	2449	-0.7
*shortchain_45	-45	1.1	9404	5.8	1564	-0.6
*shortchain_60	-60	1.0	6883	5.0	1213	-0.5

The best-fit scenario is determined using the smallest root mean square error (RMSE) to the surface ocean pH based on boron isotopes. The RMSE is calculated in R using the function "rmse" within the package "Metrics".

Table S4. Summary of the RMSE values for model-data comparison shown in Fig. S19.

Source $\delta^{13}C$ value (‰)	RMSE (pH scenario 1 Jurikova et al. (43))	RMSE (pH scenario 2 Jurikova et al. (43))	RMSE (SST based on $\delta^{18}O$ of brachiopods reported in (44))	RMSE (SST based on $\delta^{18}O$ of conodonts compiled in (45))
-9	0.3781	0.4751	4.5576	**7.2913**
**-15**	**0.3474**	**0.4616**	**3.5917**	7.4643
-18	0.3476	0.4653	3.6533	7.5802
-25	0.3522	0.4743	3.9212	7.7830
-30	0.3559	0.4794	4.0950	7.8845
-45	0.3639	0.4896	4.4562	8.0725
-60	0.3690	0.4956	4.6660	8.1765

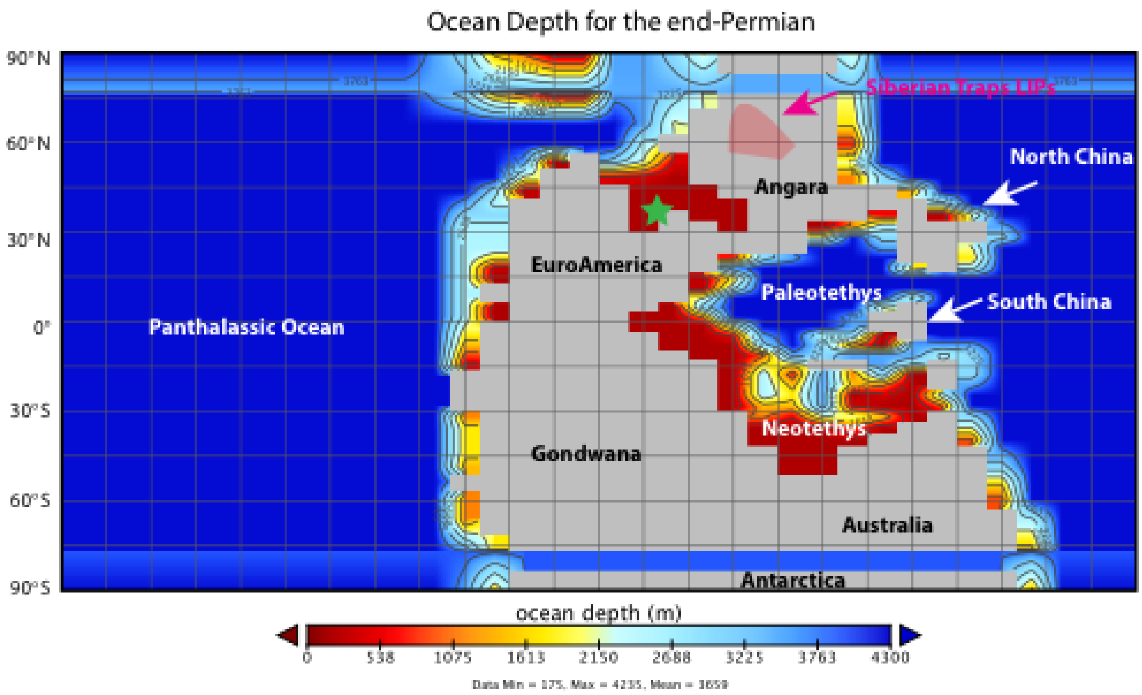

Figure S16. End-Permian ocean bathymetry used in cGENIE model. Green star shows the location of the studied site.

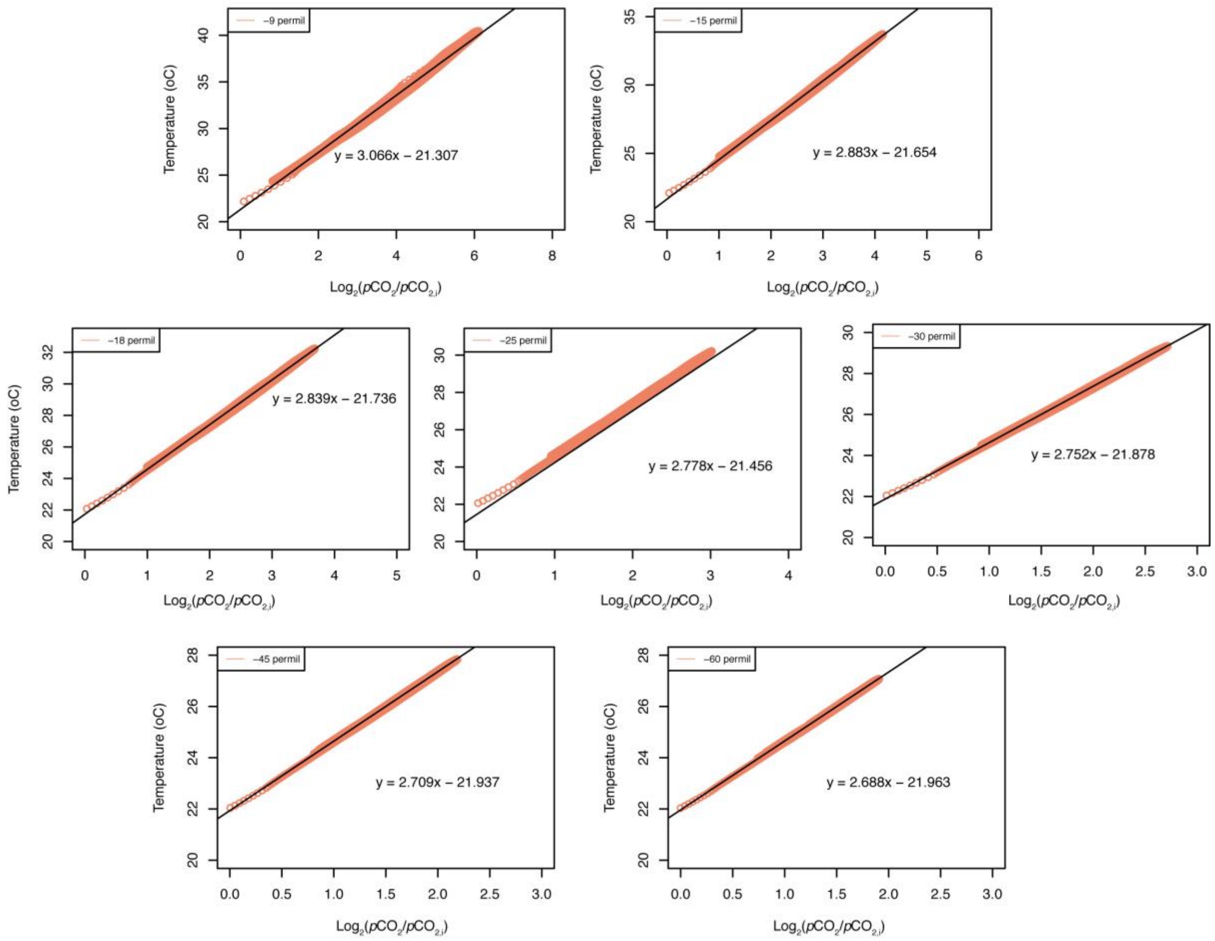

Figure S17. Calculated climate sensitivity for all seven scenarios in the isotope inversion models. Climate sensitivity (i.e. temperature change in response to a doubling of pCO_2) in cGenie is indicated by the slope of the curve.

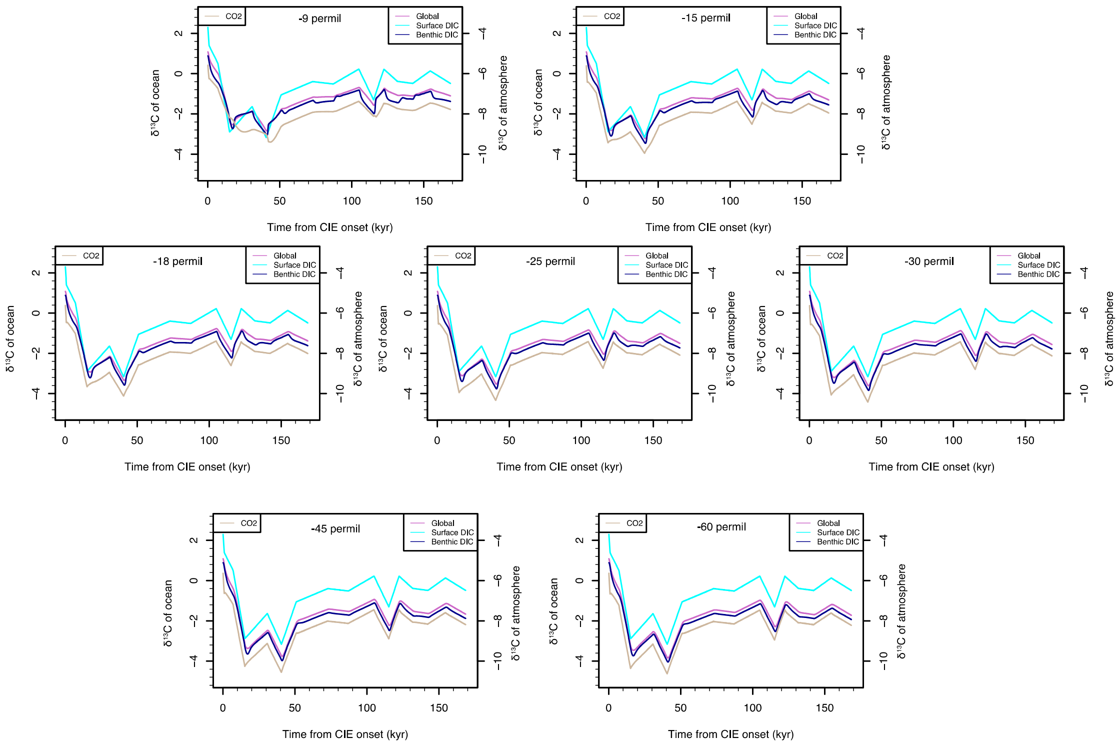

Figure S18. Modeled $\delta^{13}C$ of the atmosphere (beige), surface ocean (cyan), and deep ocean (dark blue), and the global average (purple) for all seven model scenarios ($\delta^{13}C_{source}$ = -60‰, -45‰, -30‰, -25‰, -18‰, -15‰, and -9‰).

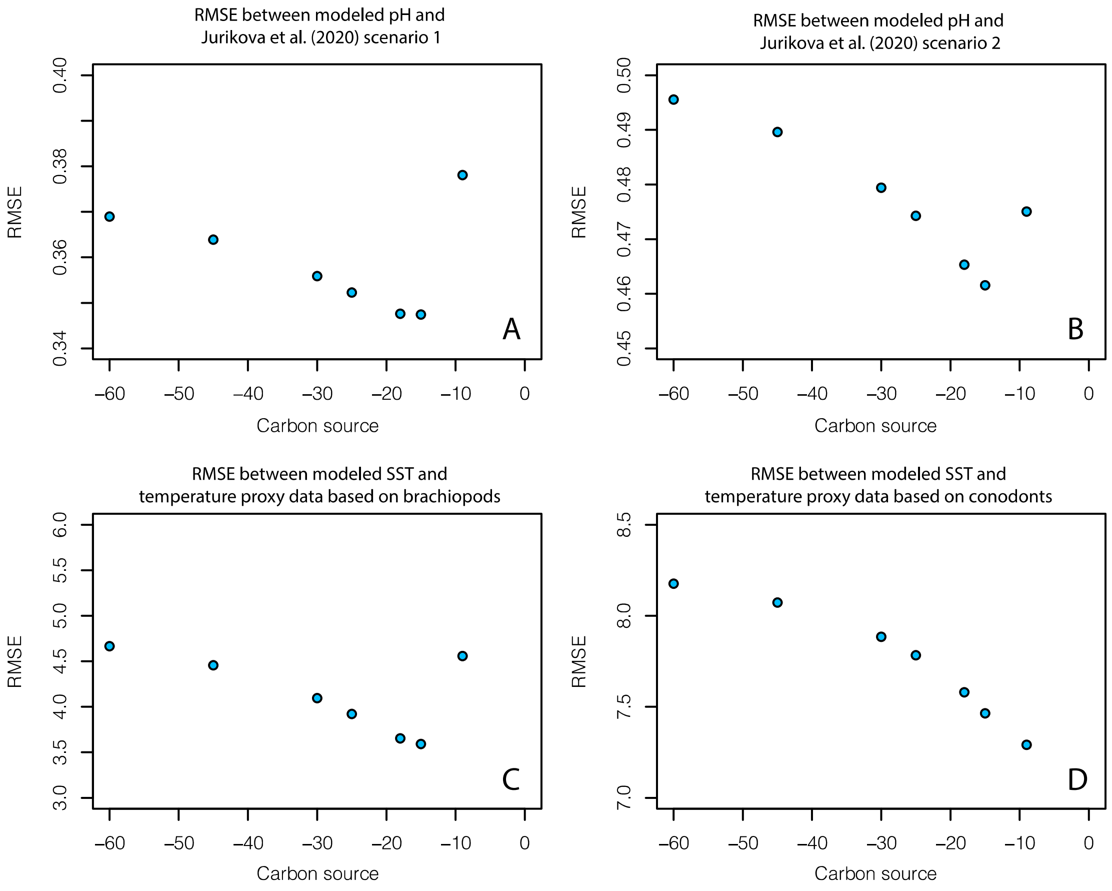

Figure S19. Root mean square error for the seven model scenarios by comparing the modeled pH (Fig. S23) and the proxy pH (panel A and B for their scenario 1 and scenario 2 in Jurikova et al. (43), and by comparing the modeled sea surface temperature (SST) and the proxy-based SST (panel C: oxygen isotope SST proxy based on well-preserved brachiopods; panel D: oxygen isotope SST proxy based on conodonts). Clarkson et al. (46) pH data are not included in the RMSE calculation because of the minimum pH does not align with Jurikova et al. (43). The seven carbon sources used in this study from left to right: $\delta^{13}C_{source}$ = -60‰, -45‰, -30‰, -25‰, -18‰, -15‰, and -9‰. The model-data comparison suggests the carbon source associated with smallest RMSE is $\delta^{13}C_{source}$ =-15‰, and therefore is considered the best-fit scenario.

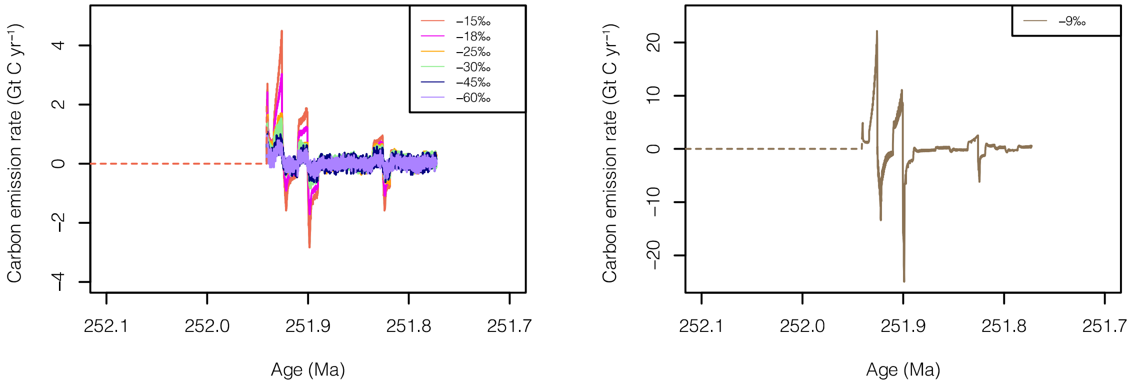

Figure S20. Modeled carbon emission rates for the seven scenarios ($\delta^{13}C_{source}$ = -60‰, -45‰, -30‰, -25‰, -18‰, -15‰). Carbon emission flux for -9‰ is shown separately to exhibit the details. The dashed line represents the equilibrium condition of the 200 kyr-long spinups.

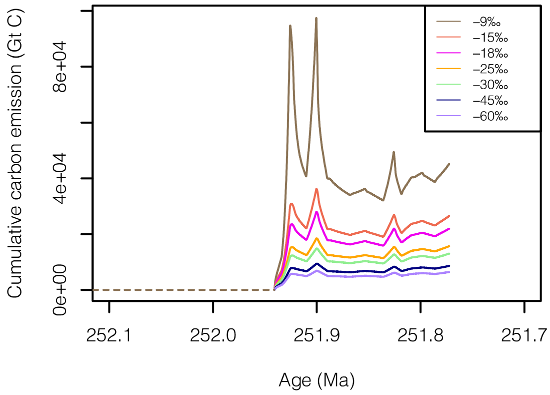

Figure S21. Modeled cumulative carbon emission amount for the seven scenarios ($\delta^{13}C_{source}$ = -60‰, -45‰, -30‰, -25‰, -18‰, -15‰, and -9‰). The dashed line represents the equilibrium condition of the 200 kyr-long spinups.

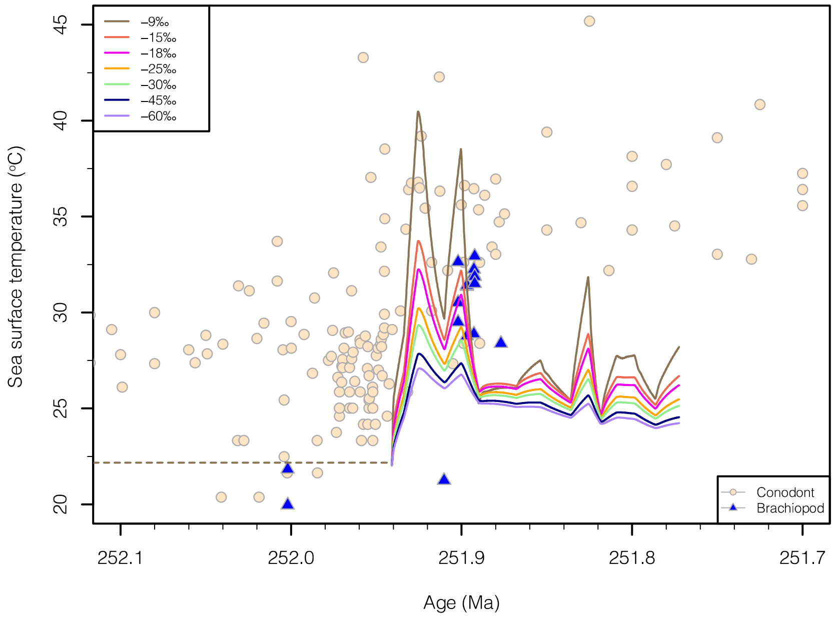

Figure S22. Modeled surface ocean temperature for the seven scenarios ($\delta^{13}C_{source}$ = -60‰, -45‰, -30‰, -25‰, -18‰, -15‰, and -9‰) and comparison to the observed temperature

proxy data based on conodonts (45) and brachiopods (44). The dashed line represents the equilibrium condition of the 200 kyr-long spinups.

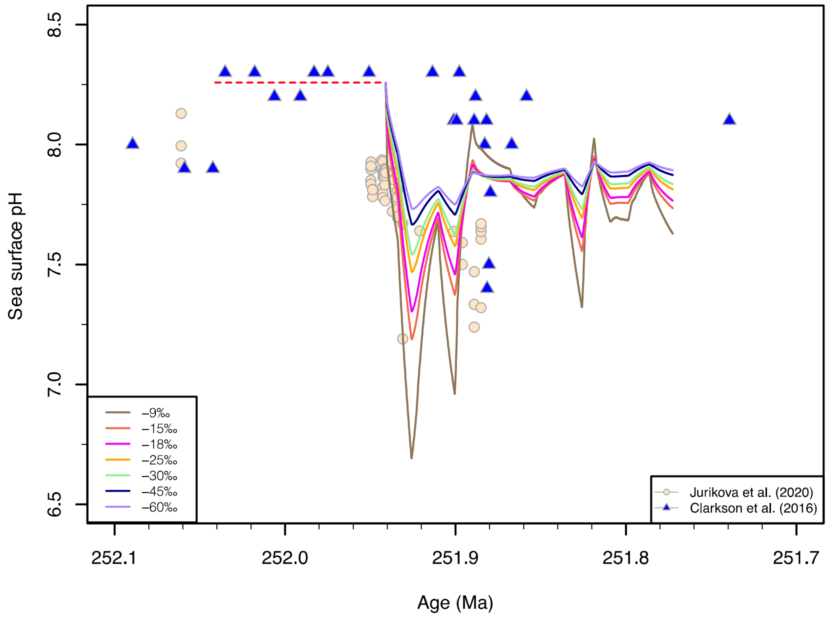

Figure S23. Modeled surface ocean pH for the seven scenarios ($\delta^{13}C_{source}$ = -60‰, -45‰, -30‰, -25‰, -18‰, -15‰, and -9‰) and the comparison to the observed data in (43) and (46). The dashed line represents the equilibrium condition of the 200 kyr-long spinups.

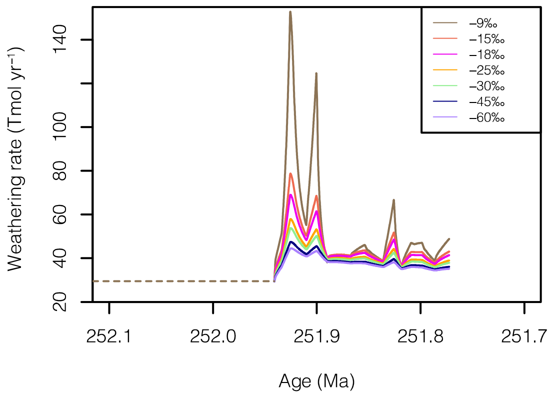

Figure S24. Modeled global weathering rate changes for the seven scenarios ($\delta^{13}C_{source}$ = -60‰, -45‰, -30‰, -25‰, -18‰, -15‰, and -9‰). The dashed line represents the equilibrium condition of the 200 kyr-long spinups.

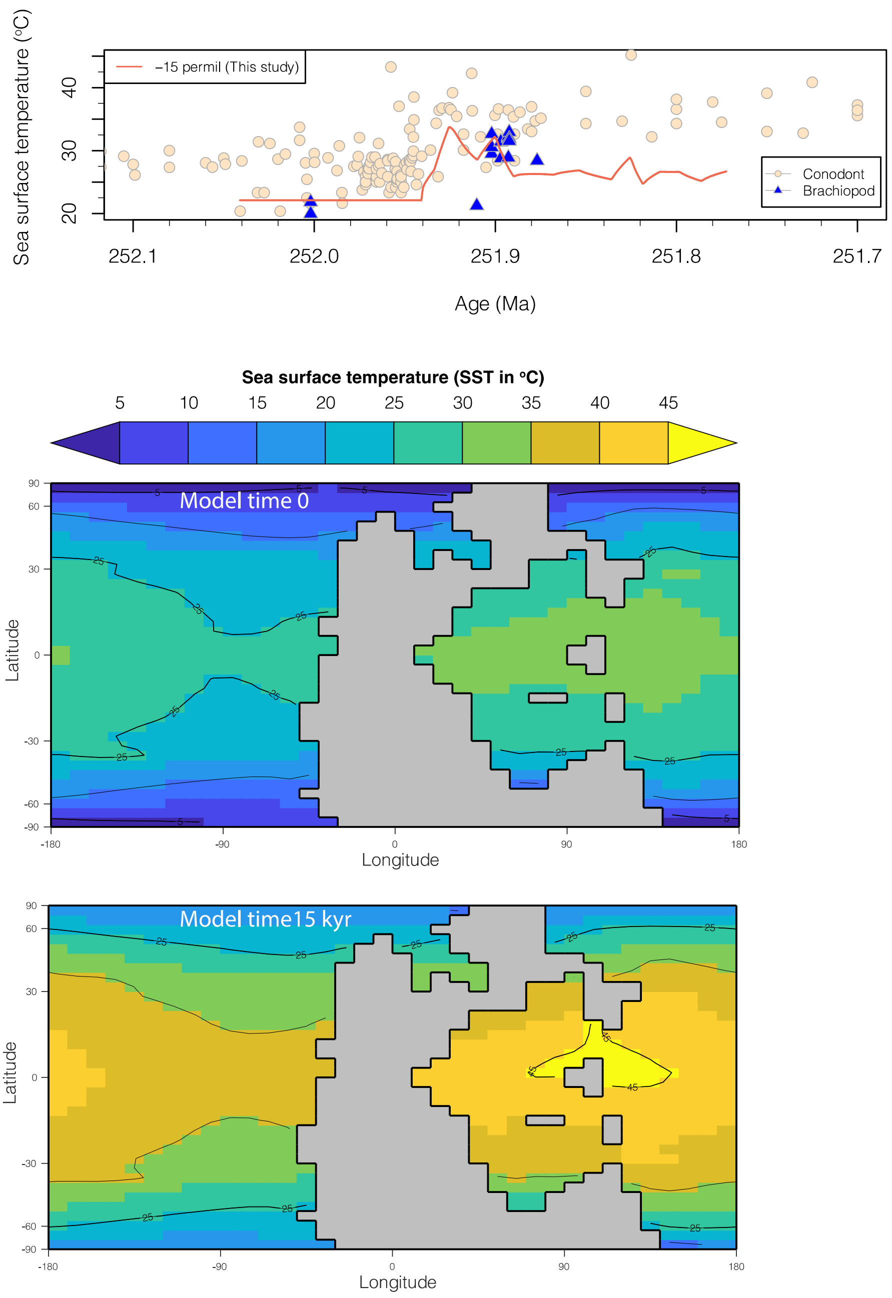

Figure S25. Spatial distribution and temporal evolution of sea surface temperature in the Earth system model of intermediate complexity cGENIE across the end-Permian mass extinction.

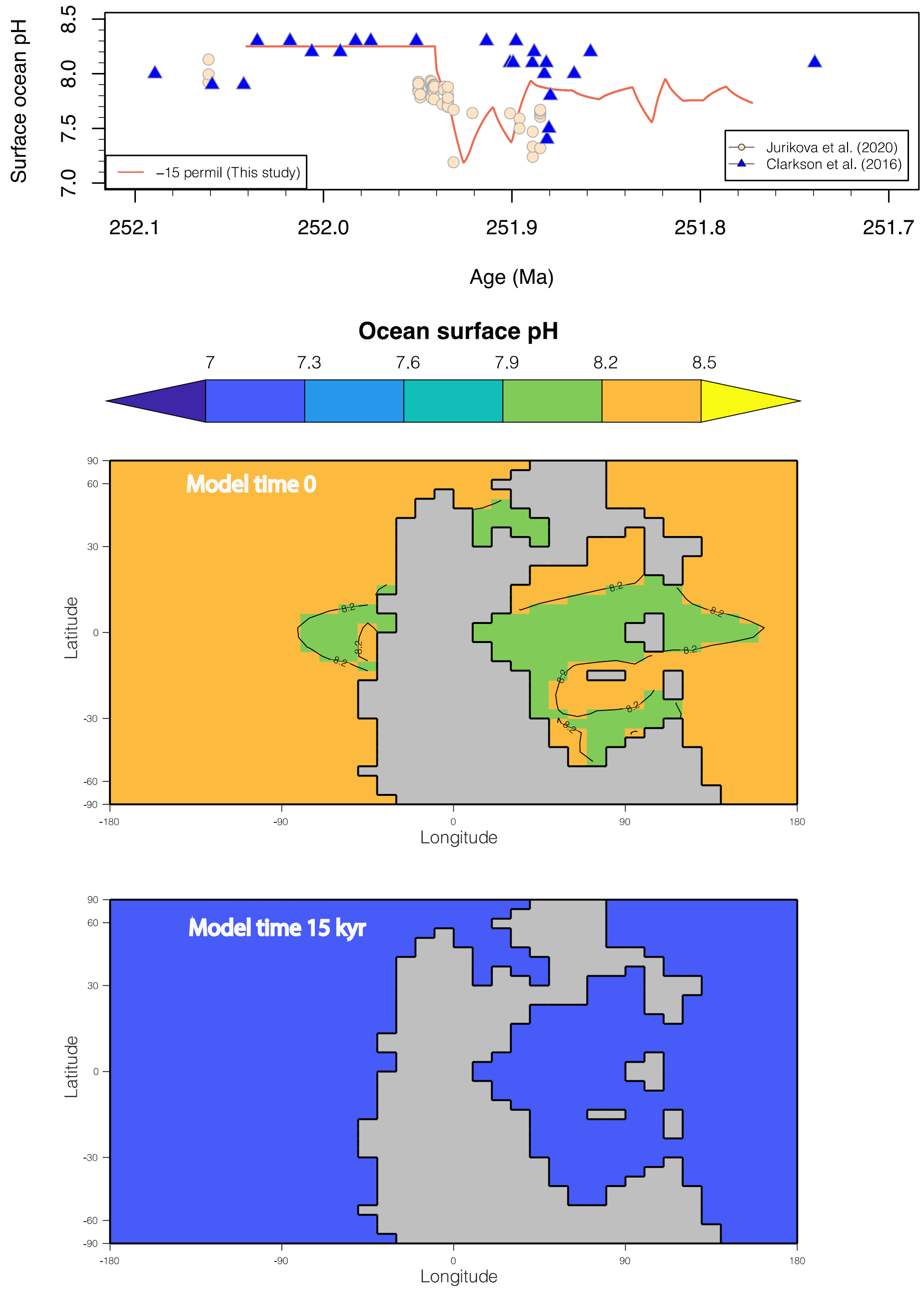

Figure S26. Spatial distribution and temporal evolution of sea surface pH in the Earth system model of intermediate complexity cGENIE across the end-Permian mass extinction. Also shown is the time-series pH from best-fit model and proxy data.

Table S5. Carbon emission estimated for the Siberian Traps.

Event	Emission Duration (years)	Emission Flux (Gt C yr^{-1})	Total Emission (Gt C)	Reference
Phreatomagmatic pipes degassing	6400	0.22 to 0.59	1400 to 3770	(47)
Contact aureoles	50,000	0.19 to 0.55	9550 to 27,270	(47)
Lava degassing	1×10^6	0.017 to 0.024	17,000 to 24,000	(47)
Intrusive and extrusive activities	1×10^6 to 2×10^6	0.01 to 0.09	19,800 to 88,000	(48)
Mantle plume source	$< 1 \times 10^6$	0.05	46,360	(49)
Pulsed sill emplacement	NA	NA	19,090 to 32,730	(50)

Table S6. Carbon isotopes of the gas emission estimated for the Siberian Traps.

Material	Source	$\delta^{13}C$ (‰)	Reference
Melt inclusions	Recycled crust component	-12 to -29	(51)
Fumarole	Mantle wedge, limestone and organic carbon (both slab and crust derived)	-5.5 to -1.3	(52)
Bubble gas from hot springs	Mantle source and recycled crustal materials	-9 to -2	(53)
Fumarolic emissions at Favare and Lake of Venus	Mantle source	-5.6 to -4.0	(54)
Pulsed degassing	Pulsed sill emplacement	-35 for TOC-derived CO_2; 0 for carbonate-derived CO_2	(50)

References

1. P. A. Hochuli, J. O. Vigran, E. Hermann, H. Bucher, Multiple climatic changes around the Permian-Triassic boundary event revealed by an expanded palynological record from mid-Norway. *Geological Society of America Bulletin* **122**, 884-896 (2010).
2. T. Bugge *et al.*, The Upper Palaeozoic succession on the Finnmark Platform, Barents Sea. *Norsk Geologisk Tidsskrift* **75**, 3-30 (1995).
3. G. Mangerud, Palynostratigraphy of the Permian and lowermost Triassic succession, Finnmark Platform, Barents Sea. *Review of Palaeobotany and Palynology* **82**, 317-349 (1994).
4. P. A. Hochuli, E. Hermann, J. O. Vigran, H. Bucher, H. Weissert, Rapid demise and recovery of plant ecosystems across the end-Permian extinction event. *Global and Planetary Change* **74**, 144-155 (2010).
5. C. H. Eide, T. G. Klausen, D. Katkov, A. A. Suslova, W. Helland-Hansen, Linking an Early Triassic delta to antecedent topography: Source-to-sink study of the southwestern Barents Sea margin. *Geological Society of America Bulletin* **130**, 263-283 (2018).
6. P. A. Hochuli, E. Hermann, J. O. Vigran, H. Bucher, H. Weissert, Rapid demise and recovery of plant ecosystems across the end-Permian extinction event. *Global and Planetary Change* **74**, 144-155 (2010).
7. A. S. Porter, C. Evans-Fitz.Gerald, C. Yiotis, I. P. Montañez, J. C. McElwain, Testing the accuracy of new paleoatmospheric CO_2 proxies based on plant stable carbon isotopic composition and stomatal traits in a range of simulated paleoatmospheric O_2:CO_2 ratios. *Geochimica et Cosmochimica Acta* https://doi.org/10.1016/j.gca.2019.05.037 (2019).
8. A. S. Porter, C. Yiotis, I. P. Montañez, J. C. McElwain, Evolutionary differences in Δ13C detected between spore and seed bearing plants following exposure to a range of atmospheric O2: CO2 ratios; implications for paleoatmosphere reconstruction. *Geochimica et Cosmochimica Acta* **213**, 517-533 (2017).
9. C. V. Looy, R. J. Twitchett, D. L. Dilcher, J. H. A. Van Konijnenburg-Van Cittert, H. Visscher, Life in the end-Permian dead zone. *Proceedings of the National Academy of Sciences* **98**, 7879-7883 (2001).
10. A. F. Diefendorf, K. E. Mueller, S. L. Wing, P. L. Koch, K. H. Freeman, Global patterns in leaf ^{13}C discrimination and implications for studies of past and future climate. *Proceedings of the National Academy of Sciences* **107**, 5738-5743 (2010).
11. M. J. Kohn, Carbon isotope compositions of terrestrial C3 plants as indicators of (paleo) ecology and (paleo) climate. *Proceedings of the National Academy of Sciences* **107**, 19691-19695 (2010).
12. B. A. Schubert, A. H. Jahren, The effect of atmospheric CO_2 concentration on carbon isotope fractionation in C3 land plants. *Geochimica et Cosmochimica Acta* **96**, 29-43 (2012).
13. K. Schlanser *et al.*, On geologic timescales, plant carbon isotope fractionation responds to precipitation similarly to modern plants and has a small negative correlation with pCO2. *Geochimica et Cosmochimica Acta* (2019).
14. Y. Cui, L. R. Kump, A. Ridgwell, Initial assessment on the carbon emission rate and climatic consequences during the end-Permian mass extinction. *Palaeogeography, Palaeoclimatology, Palaeoecology* **387**, 176-184 (2013).

15. J. L. Payne *et al.*, Large perturbations of the carbon cycle during recovery from the end-Permian extinction. *Science* **305**, 506-509 (2004).
16. L. A. Cernusak *et al.*, Environmental and physiological determinants of carbon isotope discrimination in terrestrial plants. *New Phytologist* **200**, 950-965 (2013).
17. Y. Cui, B. A. Schubert, Quantifying uncertainty of past pCO_2 determined from changes in C_3 plant carbon isotope fractionation. *Geochimica et Cosmochimica Acta* **172**, 127-138 (2016).
18. H. Wu *et al.*, Time-calibrated Milankovitch cycles for the late Permian. *Nature communications* **4**, 2452 (2013).
19. M. Li *et al.*, Astronomical-cycle scaling of the end-Permian extinction and the Early Triassic Epoch of South China and Germany. *Earth and Planetary Science Letters* **441**, 10-25 (2016).
20. M. Li, L. Hinnov, L. Kump, Acycle: Time-series analysis software for paleoclimate research and education. *Computers & Geosciences* **127**, 12-22 (2019).
21. G. P. Weedon, *Time series analysis and cyclostratigraphy: Examining stratigraphic records of environmental cycles* (Cambridge University Press, 2003), pp. 259.
22. W. S. Cleveland, LOWESS: A program for smoothing scatterplots by robust locally weighted regression. *The American Statistician* **35**, 54-54 (1981).
23. D. J. Thomson, Spectrum estimation and harmonic analysis. *Proceedings of the IEEE* **70**, 1055-1096 (1982).
24. M. E. Mann, J. M. Lees, Robust estimation of background noise and signal detection in climatic time series. *Climatic Change* **33**, 409-445 (1996).
25. K. P. Kodama, L. Hinnov, *Rock Magnetic Cyclostratigraphy* (Wiley-Blackwell, 2015), pp. 176.
26. M. Li, L. R. Kump, L. A. Hinnov, M. E. Mann, Tracking variable sedimentation rates and astronomical forcing in Phanerozoic paleoclimate proxy series with evolutionary correlation coefficients and hypothesis testing. *Earth and Planetary Science Letters* **501**, 165-179 (2018).
27. S. R. Meyers, The evaluation of eccentricity - related amplitude modulation and bundling in paleoclimate data: An inverse approach for astrochronologic testing and time scale optimization. *Paleoceanography* 10.1002/2015PA002850 (2015).
28. S. D. Burgess, S. Bowring, S.-z. Shen, High-precision timeline for Earth's most severe extinction. *Proceedings of the National Academy of Sciences of the United States of America* **111**, 3316-3321 (2014).
29. J. Laskar *et al.*, A long-term numerical solution for the insolation quantities of the Earth. *Astronomy & Astrophysics* **428**, 261-285 (2004).
30. J. Laskar, A. Fienga, M. Gastineau, H. Manche, La2010: a new orbital solution for the long-term motion of the Earth. *Astronomy & Astrophysics* **532** (2011).
31. A. Berger, M.-F. Loutre, V. Dehant, Pre-quaternary Milankovitch frequencies. *Nature*, 133 (1989).
32. M. T. Taner, *Attributes revisited* (Technical Publication, Rock Solid Images, Inc., Houston, Texas, 2000).
33. S.-z. Shen *et al.*, Calibrating the end-Permian mass extinction. *Science* **334**, 1367-1372 (2011).
34. K. Zhang, X. Lai, J. Tong, H. Jiang, Progresses on study of conodont sequence for the GSSP section at Meishan, Changxing, Zhejiang Province, South China. *Acta Palaeontologica Sinica* **48**, 474-486 (2009).

35. G. Colbourn, A. Ridgwell, T. Lenton, The Rock Geochemical Model (RokGeM) v0. 9. *Geoscientific Model Development Discussions* **5**, 2007-2082 (2012).
36. L. Cao *et al.*, The role of ocean transport in the uptake of anthropogenic CO2. *Biogeosciences* **6**, 375-390 (2009).
37. N. R. Edwards, R. Marsh, Uncertainties due to transport-parameter sensitivity in an efficient 3-D ocean-climate model. *Climate Dynamics* **24**, 415-433 (2005).
38. J. Hargreaves, J. Annan, N. Edwards, R. Marsh, An efficient climate forecasting method using an intermediate complexity Earth System Model and the ensemble Kalman filter. *Climate Dynamics* **23**, 745-760 (2004).
39. A. Ridgwell, J. C. Hargreaves, Regulation of atmospheric CO_2 by deep-sea sediments in an Earth system model. *Global Biogeochemical Cycles* **21**, GB2008 (2007).
40. A. Ridgwell, Interpreting transient carbonate compensation depth changes by marine sediment core modeling. *Paleoceanography* **22**, PA4102 (2007).
41. A. Ridgwell *et al.*, Marine geochemical data assimilation in an efficient Earth System Model of global biogeochemical cycling. **4**, 87-104 (2007).
42. H. Li, J. Yu, J. C. McElwain, C. Yiotis, Z.-Q. Chen, Reconstruction of atmospheric CO2 concentration during the late Changhsingian based on fossil conifers from the Dalong Formation in South China. *Palaeogeography, Palaeoclimatology, Palaeoecology* **519**, 37-48 (2019).
43. H. Jurikova *et al.*, Permian–Triassic mass extinction pulses driven by major marine carbon cycle perturbations. *Nature Geoscience* 10.1038/s41561-020-00646-4 (2020).
44. W.-q. Wang *et al.*, A high-resolution Middle to Late Permian paleotemperature curve reconstructed using oxygen isotopes of well-preserved brachiopod shells. *Earth and Planetary Science Letters* **540**, 116245 (2020).
45. Y. Wu *et al.*, Six-fold increase of atmospheric pCO_2 during the Permian–Triassic mass extinction. *Nature communications* **12**, 1-8 (2021).
46. M. O. Clarkson *et al.*, Ocean acidification and the Permo-Triassic mass extinction. *Science* **348**, 229-232 (2015).
47. H. Svensen *et al.*, Siberian gas venting and the end-Permian environmental crisis. *Earth and Planetary Science Letters* **277**, 490-500 (2009).
48. A. D. Saunders, Two LIPs and two Earth-system crises: the impact of the North Atlantic Igneous Province and the Siberian Traps on the Earth-surface carbon cycle. *Geological Magazine* **153**, 201-222 (2016).
49. S. V. Sobolev *et al.*, Linking mantle plumes, large igneous provinces and environmental catastrophes. *Nature* **477**, 312-316 (2011).
50. H. H. Svensen *et al.*, Sills and gas generation in the Siberian Traps. *Philosophical Transactions of the Royal Society A: Mathematical, Physical and Engineering Sciences* **376**, 20170080 (2018).
51. E. Hauri, SIMS analysis of volatiles in silicate glasses, 2: isotopes and abundances in Hawaiian melt inclusions. *Chemical Geology* **183**, 115-141 (2002).
52. D. R. Hilton *et al.*, Monitoring of temporal and spatial variations in fumarole helium and carbon dioxide characteristics at Poás and Turrialba volcanoes, Costa Rica (2001-2009). *Geochemical Journal* **44**, 431-440 (2010).
53. M. Zhang *et al.*, Magma-derived CO2 emissions in the Tengchong volcanic field, SE Tibet: implications for deep carbon cycle at intra-continent subduction zone. *Journal of Asian Earth Sciences* **127**, 76-90 (2016).

54. R. Favara, S. Giammanco, S. Inguaggiato, G. Pecoraino, Preliminary estimate of CO2 output from Pantelleria Island volcano (Sicily, Italy): evidence of active mantle degassing. *Applied Geochemistry* **16**, 883-894 (2001).

Table S7. Organic geochemistry data used in figures.

Sample	depth (m)	Time (Ma) PTB = 251.941 Ma	C17	C18	C19	C20	C21	C22	C23	C24	C25	C26	C27	C28	C29	C30	C31	C33	C35
**core 7128/12-U-01**																			
B1	79.4	251.7638				-32.98	-34.13	-33.85	-34.07	-34.25	-33.52	-35.84	-35.76	-35.58	-37.21	-36.67	-36.70	-36.69	-36.46
B2	81.3	251.7723		-31.62	-31.67	-31.91	-33.63	-33.51	-34.05	-33.79	-34.40	-36.72	-36.39	-39.22	-37.27	-38.00	-36.62	-37.18	-35.67
B3	84.5	251.7865	-31.91	-31.91	-30.17	-31.98	-32.22	-32.87	-32.71	-33.31	-33.52	-34.02	-35.78	-35.63	-37.57	-37.75	-36.34	-35.46	-36.62
B4	87.2	251.7986	-31.66	-33.85		-34.62	-35.95	-35.14	-35.49	-35.37	-32.72	-34.92	-36.16	-35.73	-36.83	-36.02	-36.11	-35.87	-37.00
B5	89.6	251.8093		-32.38	-31.56	-32.60	-33.95	-34.95	-35.50	-35.18	-31.87	-35.55	-35.83	-37.55	-36.66	-38.10	-35.50	-36.54	-35.47
B6	91.7	251.8187		-33.74	-30.95	-34.85	-35.47	-35.06	-35.63	-35.38	-35.29	-35.55	-36.77	-36.25	-36.83	-36.48	-36.64	-36.32	-36.12
B7	93.3	251.8258	-32.12	-32.49	-32.83	-33.83	-35.78	-34.54	-35.56	-37.20	-32.38	-36.60	-36.23	-36.98	-37.04	-37.05	-36.66	-36.59	-36.15
B8	95.6	251.8361	-31.56	-32.07	-30.33	-31.21	-31.82	-32.96	-33.40	-30.81	-31.57	-32.25	-32.03		-35.09		-37.03	-33.61	
B9	99.6	251.8539	-31.59	-31.68	-31.79	-34.31	-32.86	-33.16	-33.64	-32.81		-34.22	-34.17	-32.73	-35.05	-35.53	-35.91	-33.89	-35.51
B10	102.8	251.8682	-32.52	-31.96	-30.62	-31.86	-31.86	-32.38	-32.51	-31.60	-31.62	-32.10	-33.82	-34.74	-36.92	-35.60	-37.71	-35.11	-38.89
B11	104.7	251.8767									-34.26		-35.67		-36.53		-36.77	-35.75	-34.92
B12	107.7	251.8901	-32.54	-33.16	-31.92	-34.56		-34.35	-33.69	-34.26			-35.51		-36.87		-37.12	-35.95	-36.44
B13	110	251.9004	-34.18	-35.12	-34.50	-34.05	-34.03	-34.28	-34.51	-34.67	-31.66	-35.98	-35.35	-33.39	-36.05	-35.55	-36.31	-36.10	-32.14
B14	112.2	251.9102	-34.38	-34.35	-31.24	-32.11	-33.61	-33.29	-33.73	-33.93	-32.77	-34.86	-35.63	-36.19	-36.54	-36.38	-36.94	-36.23	-36.91
B15	115.7	251.9258	-35.53	-34.61	-32.60	-33.76	-33.65	-32.94	-34.39	-32.65					-38.49		-38.43		
B21	139.8	252.0334	-28.52	-28.92	-28.94	-29.12	-29.65	-29.62	-29.01	-28.89	-29.45	-28.54	-25.19	-28.05	-25.07	-27.63	-31.49	-27.97	-38.64
B16	141.8	252.0423	-30.20	-30.36	-31.26	-31.22	-30.31	-30.36	-27.93	-29.00	-29.60	-27.70	-25.18		-26.96		-32.84		
B22	143.6	252.0504	-28.48	-28.90	-28.93	-28.54	-28.76	-28.62	-28.98	-28.49	-28.39	-27.61	-25.75	-29.20	-28.72	-30.53	-33.53	-31.86	
B23	145.8	252.0602	-29.92	-29.89	-30.50	-30.26	-30.63	-31.37	-29.92	-30.23	-30.12	-29.79	-27.31	-28.56	-27.75	-29.18	-33.70	-30.40	
**core 7129/10-U-01**																			
B17 (64.5)	117.5	251.9339	-30.30	-31.17	-31.03	-31.19	-31.79	-31.93	-31.86	-32.28	-31.08	-31.91	-31.36	-32.77	-32.62	-33.22	-32.12	-32.40	-33.18
B18 (65.9)	118.9	251.9401	-30.33	-30.89	-29.23	-29.54	-30.02	-30.51	-31.60	-30.18		-31.38	-28.96	-30.05	-33.20		-29.20	-32.37	-29.90
B19 (66.1)	119.1	251.9410	-29.46	-29.25	-28.06	-27.93	-28.45	-27.53	-27.78	-27.67	-28.72	-27.19	-29.44	-26.83	-32.34	-28.88	-34.63	-31.54	

Table S7 (continued). Organic geochemistry data used in figures.

Sample	depth (m)	Time (Ma)	av. C17,19	C17,18,19	CPI	ACL 25-33	C31/27	C17/C27	Pr/Ph	d13CV-PDB
**core 7128/12-U-01**		PTB = 251.941 Ma								
B1	79.4	251.7638			3.64	29.23	1.59	0.00	0.58	-30.34
B2	81.3	251.7723	-31.67	-31.64	3.23	29.24	1.51	0.13	0.81	-30.95
B3	84.5	251.7865	-31.04	-31.33	3.53	28.91	1.05	0.34	1.33	-31.12
B4	87.2	251.7986	-31.66	-32.75	3.18	28.93	1.12	0.40	0.81	-29.57
B5	89.6	251.8093	-31.56	-31.97	3.11	29.01	1.11	0.13	1.08	-29.71
B6	91.7	251.8187	-30.95	-32.35	3.38	29.12	1.38	0.13	0.82	-30.97
B7	93.3	251.8258	-32.48	-32.48	3.43	29.43	1.74	1.30	1.38	-29.69
B8	95.6	251.8361	-30.95	-31.32	2.45	29.42	2.21	1.65	1.18	-27.65
B9	99.6	251.8539	-31.69	-31.69	3.49	29.54	2.05	0.87	1.06	-28.76
B10	102.8	251.8682	-31.57	-31.70	3.11	29.33	2.47	1.01	0.69	-28.46
B11	104.7	251.8767			3.00	29.40	1.71	0.20	0.77	-28.33
B12	107.7	251.8901	-32.23	-32.54	3.20	29.18	1.86	0.92	0.96	-27.84
B13	110	251.9004	-34.34	-34.60	2.33	28.56	0.69	1.38	1.51	-28.59
B14	112.2	251.9102	-32.81	-33.32	2.98	28.96	1.07	1.71	0.91	-29.34
B15	115.7	251.9258	-34.06	-34.24	2.93	28.56	0.83	1.32	0.61	-27.44
B21	139.8	252.0334	-28.73	-28.80	2.30	27.91	0.92	0.63	0.79	-23.35
B16	141.8	252.0423	-30.73	-30.61	2.66	29.20	0.40	0.72	1.15	-23.33
B22	143.6	252.0504	-28.70	-28.77	2.09	28.26	1.34	4.52	0.80	-24.38
B23	145.8	252.0602	-30.21	-30.10	2.04	27.75	0.72	0.71	0.60	-23.22
**core 7129/10-U-01**										
B17 (64.5)	117.5	251.9339	-30.67	-30.84	2.66	29.20	0.92	0.63	1.10	-27.99
B18 (65.9)	118.9	251.9401	-29.78	-30.15	2.41	28.56	1.69	0.91	0.95	-26.29
B19 (66.1)	119.1	251.9410	-28.76	-28.92	2.31	28.46	1.74	11.34	1.48	-26.16

Table S8. CSI values of repeated analyses of individual samples.

sample	depth (m)	C17 A	C17 B	C17C	C17 D	**C17average**	**stdev**	C19 A	C19 B	C19C	C19 D	**C19average**	**stdev**	**averageC17,19**	**stdev**	**weighed average**	**stdev**
**core 7128/12-U-01**																	
B1	79.4																
B2	81.3								-31.7			-31.7		-31.7		-31.7	
B3	84.5	-32.2	-31.6			-31.9	0.4		-30.2			-30.2		-31.0	1.2	-31.3	1.1
B4	87.2	-32.0	-31.3			-31.7	0.4							-31.7		-31.7	0.4
B5	89.6								-31.6	-31.5		-31.6	0.1	-31.6		-31.6	0.1
B6	91.7								-31.0			-31.0		-31.0		-31.0	
B7	93.3	-31.7	-30.0	-33.2	-33.6	-32.1	1.7	-32.7	-31.1	-35.4	-32.2	-32.8	1.8	-32.5	0.5	-32.5	1.7
B8	95.6	-31.1	-32.0			-31.6	0.6	-30.2	-30.5			-30.3	0.2	-30.9	0.9	-30.9	0.8
B9	99.6		-31.6			-31.6			-31.8			-31.8		-31.7	0.1	-31.7	0.1
B10	102.8		-32.5			-32.5		-30.8	-30.4			-30.6	0.3	-31.6	1.3	-31.3	1.1
B11	104.7																
B12	107.7	-32.5				-32.5		-31.9				-31.9		-32.2	0.4	-32.2	0.4
B13	110.0	-34.2				-34.2		-34.5				-34.5		-34.3	0.2	-34.3	0.2
B14	112.2		-34.4			-34.4			-31.2			-31.2		-32.8	2.2	-32.8	2.2
B15	115.7	-35.0	-36.0			-35.5	0.7	-32.9	-32.3			-32.6	0.4	-34.1	2.1	-34.1	1.8
B21	139.8		-28.5			-28.5		-28.8	-29.1			-28.9	0.2	-28.7	0.3	-28.8	0.3
B16	141.8	-30.2				-30.2		-31.3				-31.3		-30.7	0.7	-30.7	0.7
B22	143.6	-28.4	-28.6			-28.5	0.1	-28.6	-29.2			-28.9	0.4	-28.7	0.3	-28.7	0.4
B23	145.8		-29.9			-29.9		-30.7	-30.3			-30.5	0.2	-30.2	0.4	-30.3	0.4
**core 7129/10-U-01**																	
B17 (64.5)	117.0	-30.3				-30.3			-31.0			-31.0		-30.7	0.5	-30.7	0.5
B18 (65.9)	118.4	-30.3				-30.3		-29.2				-29.2		-29.8	0.8	-29.8	0.8
B19 (66.1)	118.6	-29.8	-29.1			-29.5	0.5	-28.5	-27.6			-28.1	0.7	-28.8	1.0	-28.8	0.9

Table S8 (continued). CSI values of repeated analyses of individual samples

	depth (m)	C27 A	C27 B	C27C	C27 D	C27	stdev	C29 A	C29 B	C29 C	C29 D	C29	stdev
core 7128/12-U-01													
B1	79.4	-35.0	-36.5			-35.8	1.0	-37.5	-37.0			-37.2	0.4
B2	81.3	-35.8	-36.9			-36.4	0.8	-36.8	-37.7			-37.3	0.6
B3	84.5		-35.8			-35.8			-37.6			-37.6	
B4	87.2	-36.3	-36.0			-36.2	0.2	-36.8	-36.9			-36.8	0.0
B5	89.6	-35.0	-36.7			-35.8	1.2	-35.5	-37.8			-36.7	1.6
B6	91.7	-37.0	-36.6			-36.8	0.3	-36.9	-36.7			-36.8	0.2
B7	93.3	-36.3	-35.7	-35.8	-37.0	-36.2	0.6	-37.6	-36.9	-36.7	-37.0	-37.0	0.4
B8	95.6		-32.0			-32.0		-35.5	-34.7			-35.1	0.6
B9	99.6	-34.0	-34.3			-34.2	0.2	-34.6	-35.5			-35.1	0.7
B10	102.8		-33.8			-33.8		-37.9	-36.0			-36.9	1.3
B11	104.7	-35.4	-35.9			-35.7	0.3	-37.0	-36.1			-36.5	0.6
B12	107.7	-35.5				-35.5		-36.5	-37.2			-36.9	0.5
B13	110.0	-35.4				-35.4		-36.0				-36.0	
B14	112.2	-36.0	-35.3			-35.6	0.5	-36.7	-36.4			-36.5	0.2
B15	115.7								-38.5			-38.5	
B21	139.8	-25.2	-25.2			-25.2	0.0	-25.4	-24.8			-25.1	0.4
B16	141.8	-25.7	-24.7			-25.2	0.7	-27.3	-26.6			-27.0	0.5
B22	143.6	-24.5	-27.0			-25.8	1.8	-28.4	-29.0			-28.7	0.4
B23	145.8	-27.0	-27.6			-27.3	0.4	-27.9	-27.6			-27.7	0.2
core 7129/10-U-01													
B17 (64.5)	117.0	-30.8	-32.0			-31.4	0.8	-32.5	-32.7			-32.6	0.2
B18 (65.9)	118.4	-29.0				-29.0		-33.2				-33.2	
B19 (66.1)	118.6	-30.4	-28.5			-29.4	1.3	-33.4	-31.3			-32.3	1.4
